# Phlebotomine sandflies (Diptera, Psychodidae) from the Ankarana *tsingy* of northern Madagascar: inventory and description of new taxa

**DOI:** 10.1051/parasite/2019039

**Published:** 2019-06-30

**Authors:** Antoine Blavier, Laetitia Laroche, Fano José Randrianambinintsoa, Vincent Lucas, Jean-Charles Gantier, Nicole Léger, Vincent Robert, Jérôme Depaquit

**Affiliations:** 1 Université de Reims Champagne-Ardenne, Faculté de Pharmacie, ANSES, SFR Cap Santé, EA7510 ESCAPE – USC VECPAR 51 rue Cognacq-Jay 51096 Reims Cedex France; 2 MIVEGEC Unit, IRD, CNRS, Univ Montpellier 911 avenue Agropolis BP 64501 34394 Montpellier Cedex 5 France; 3 Laboratoire de Parasitologie-Mycologie, CHU de Reims 51100 Reims France

**Keywords:** Phlebotomine sandflies, Madaphlebotomus, Sergentomyia, Morphological and molecular taxonomy

## Abstract

An inventory of Phlebotomine sandflies was carried out in the Ankarana *tsingy* located in far northern Madagascar. A total of 723 sandflies were used for morphological, morphometric, and molecular studies (sequencing of partial cytochrome B (mtDNA) and partial 28S (rDNA)). Nine species were identified: *Phlebotomus fertei, Sergentomyia anka, Se. sclerosiphon, Se. goodmani*, two species of the genus *Grassomyia*, as well as three new species described herein: *Se. volfi* n. sp., *Se. kaltenbachi* n. sp., and *Se. ozbeli* n. sp. The recognition of these new species is strongly supported by molecular analyses. The first two of the new species could not be classified into any existing subgenus, therefore we proposed two new subgenera (*Ranavalonomyia* subg. nov., and *Riouxomyia* subg. nov.), with combinations as: *Sergentomyia (Ranavalonomyia) volfi* and *Sergentomyia (Riouxomyia) kaltenbachi*. Our study reveals important molecular variability in *Se. anka*, with the recognition of a population whose taxonomic status remains below that of species. Our research confirms the need to further study the specific diversity of Malagasy sandflies, which until the start of this millennium remained mostly unknown.

## Abstract (fr)

Un inventaire des Phlébotomes a été réalisé dans les *tsingy* d’Ankarana à l’extrême nord de Madagascar. Au total, 723 phlébotomes ont servi à des études morphologique, morphométrique et moléculaire (séquençage d’une partie du cytochrome B (ADNmt) et d’une partie de l’ADNr 28S). Neuf espèces ont été identifiées : *Phlebotomus fertei*, *Sergentomyia anka*, *Se. sclerosiphon*, *Se. goodmani*, deux espèces du genre *Grassomyia*, ainsi que trois espèces nouvelles décrites dans ce travail : *Se. volfi* n. sp., *Se. kaltenbachi* n. sp., and *Se. ozbeli* n. sp. L’individualisation de chacune de ces espèces nouvelles est robustement soutenue par les analyses moléculaires. Les deux premières de ces espèces nouvelles ne pouvaient pas être classées dans un sous-genre existant et nous avons proposé pour elles deux sous-genres nouveaux : *Ranavalonomyia* subg. nov., et *Riouxomyia* subg. nov, avec les combinaisons *Sergentomyia (Ranavalonomyia) volfi* et *Sergentomyia (Riouxomyia) kaltenbachi*.Notre étude révèle une variabilité moléculaire marquée chez *Se. anka* avec l’individualisation d’une population dont le statut taxinomique demeure populationnel. Nos travaux confirment la nécessité de poursuivre l’étude de la biodiversité des Phlébotomes qui est restée méconnue dans ce pays jusqu’au début de ce millénaire.

## Introduction

The diversity of Phlebotomine sandflies (Diptera, Psychodidae) of Madagascar has long been understudied because that this island country is known to be leishmaniasis-free.

Two species belonging to the genus *Grassomyia*, *Gr. squamipleuris* Newstead 1912 and *Gr. madagascariensis* Abonnenc 1969, were initially reported from Madagascar [1, 2]. In 1978, Léger and Rodhain described *Phlebotomus berentiensis* in the genus *Sergentomyia*. This sandfly with specific characteristics was moved into the genus *Phlebotomus* Rondani & Berté, according to the characteristics of the male described later [7]. The first record of *Phlebotomus* in Madagascar was made in 2002 with the description of a male *Ph. fertei* Depaquit, Léger & Robert [9]. As of today, 14 species have been identified in Madagascar, including five *Phlebotomus*: *Ph. fertei*, *Ph. berentiensis*, *Ph. fontenillei* Depaquit, Léger & Robert 2004, *Ph. vaomalalae* Randrianambinintsoa, Léger & Depaquit 2013, and *Ph. vincenti* Randrianambinintsoa & Depaquit 2013. All of them belong to the endemic subgenus *Madaphlebotomus* Depaquit, Léger & Randrianambinintsoa 2015 [8].

At the present time, the genus *Sergentomyia* includes seven species: *Se*. (*Trouilletomyia*) *huberti* (Depaquit, Léger & Robert 2002), *Se*. (*Rondanomyia*) *goodmani* Léger, Depaquit & Robert 2005, *Se. majungaensis* Depaquit, Léger & Robert 2007, *Se*. (*Vattieromyia*) *sclerosiphon* Depaquit, Léger & Robert 2008, *Se*. (*Vat.*) *namo* Depaquit, Léger & Robert 2008, *Se*. (*Vat.*) *anka* Depaquit, Léger & Robert 2008, and *Se*. (*Tro.*) *boironis* Randrianambinintsoa & Depaquit 2014 [9, 11, 12, 26, 33].

**Figure 1:**
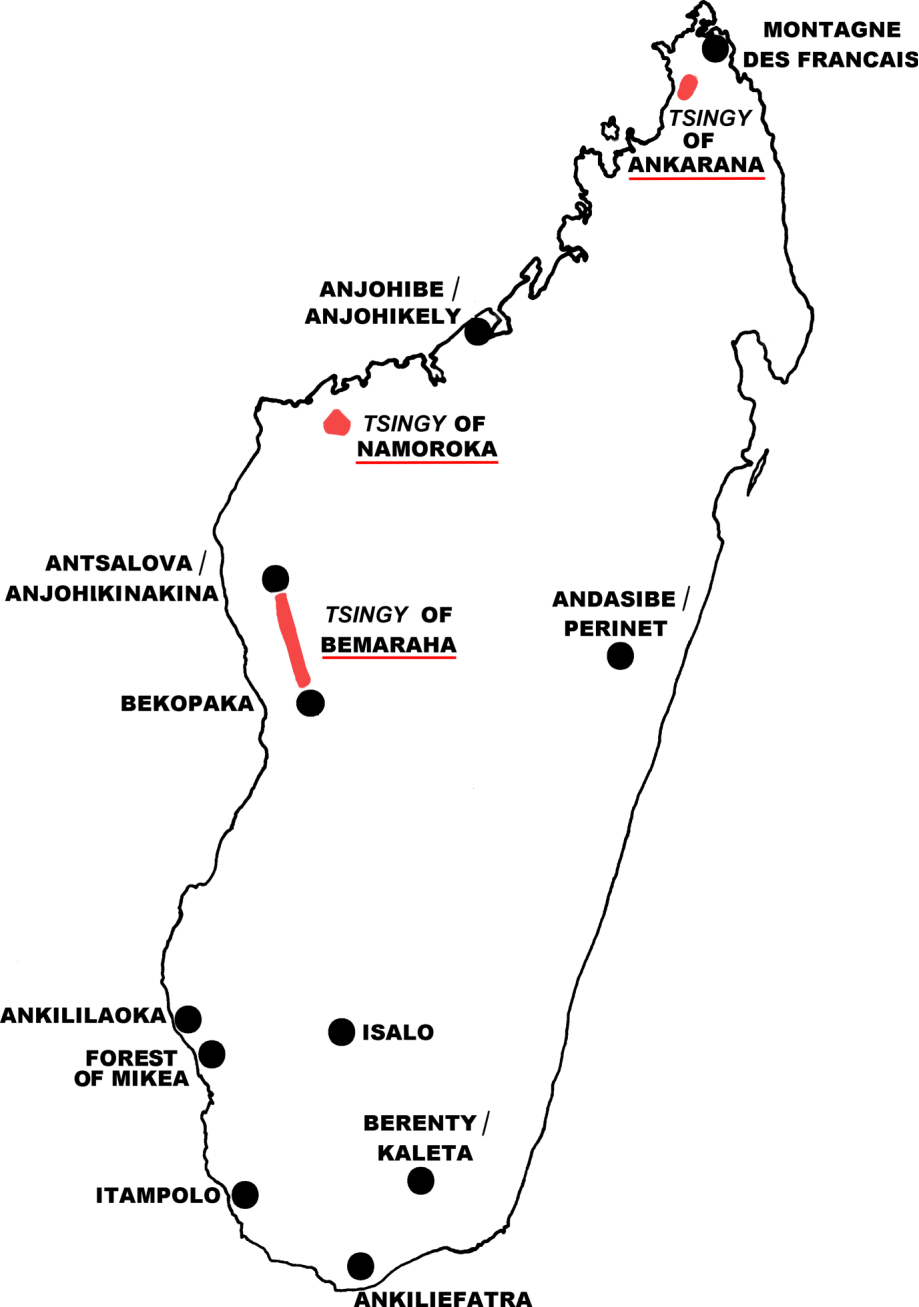
Map of Madagascar showing the *tsingy* of Ankarana, the other *tsingy*, and the places where sandflies were caught.

Our research objective was to investigate the sandflies found in the karst formations (eroded limestone spires), known as *tsingy*, of the Ankarana region in the far north of the island (Fig. 1).

For a long time, only the species of the genus *Phlebotomus*, known to take blood-meals on mammals and birds, were considered able to transmit *Leishmania* in the Old World [23]. However, the genus *Sergentomyia*, whose role was ignored for too long, cannot be neglected as a potential vector [28]. Many *Sergentomyia* species feed on reptiles [2, 17], but some of them are able to feed on mammals [4, 22, 36]. We do not know whether the Malagasy sandflies could have vectorial competence for some *Leishmania*. Some *Leishmania* could be transmitted by sandflies differing from their usual vectors in the New World as well as in the Old World [6, 16, 23, 37]. The role of Malagasy species in *Leishmania* transmission remains unknown and could be evaluated based on newly colonized species [25].

## Materials and methods

### Ethics statement

A research license for collecting and transporting zoological material (099-MEF/SG/DGEF/DADF/SCB, 5 May 2003) was obtained from Madagascar National Parks and the *Ministère des Forêts et de l’Environnement*. No endangered or protected species were collected in this study.

### Sandfly sampling

**Figure 2:**
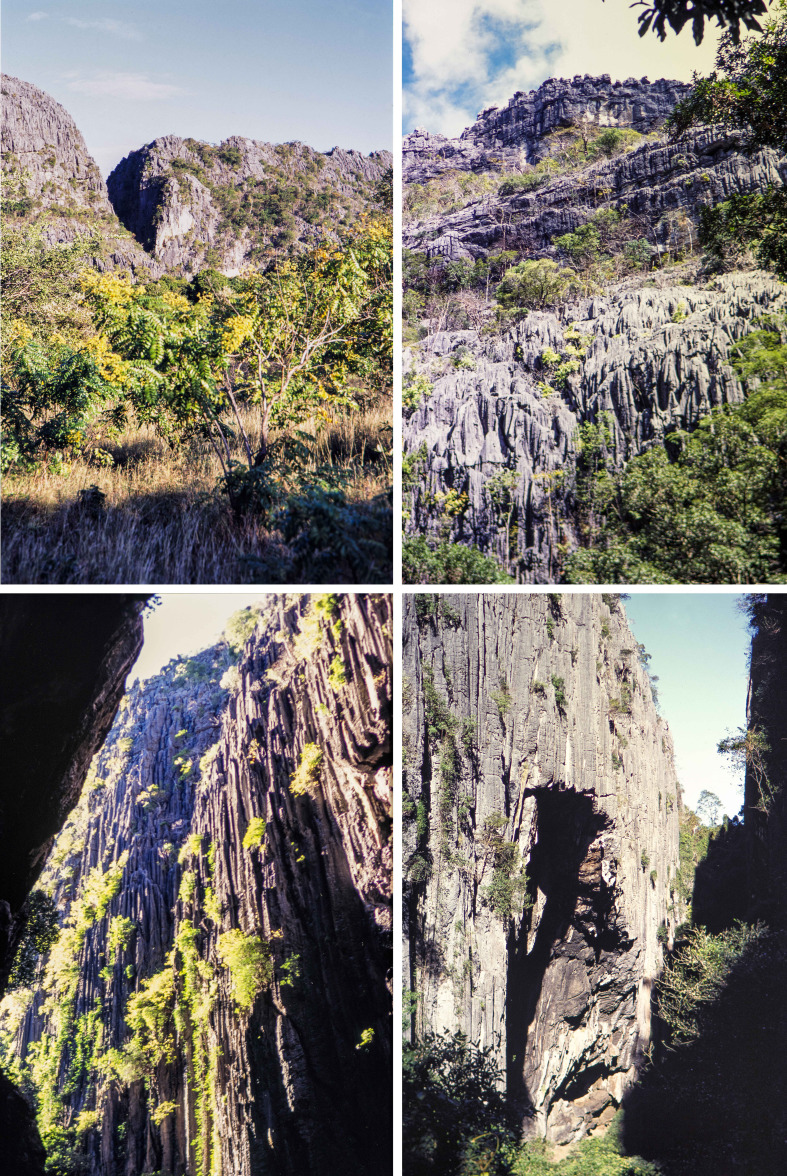
Four photos of the *Tsingy* of Ankarana taken in May 2003. Photos: Vincent Robert.

Sampling was carried out at the *Réserve Spéciale d’Ankarana*, Diana Region (ex-province of Antsiranana) in the northern part of the island, 70 km south of the town of Antsiranana. The collection site was many kilometers from any human habitation and in an area of dry deciduous forest surrounded by karst formations of eroded limestone spires, known as *tsingy*. This massif is an outcrop of middle Jurassic limestone, oriented NE-SW and of about 25 km long and 8 km wide. Quaternary earth movements (c. 1.5 million years ago) resulted in the splitting of the massif and the elevation of its western wall, which is now marked by vertical cliffs rising 100–150 m above the surrounding area. A series of volcanic eruptions in recent geological times produced lava flows around the massif, including directly into some of the canyons (0.5 million years ago). The Ankarana Massif is filled with a diversity of water cut canyons, crevices, and many caves. These different formations represent a wide variety of geomorphological types and presumably provide a considerable range of different habitats for invertebrates (Fig. 2). The Ankarana region receives slightly less than 2000 mm of rainfall annually [20]. Even with this relatively high annual precipitation, the site has dry deciduous vegetation due to a long dry season, lasting most years from May to November, and when the average daily temperature reaches about 26 °C. The rainy season is generally from December to April, and accounts for 93% of the annual precipitation, and the average daily temperature is 27.5 °C [18, 27].

Sandflies were collected over seven consecutive nights, between 20 May and 26 May 2003. They were captured using CDC miniature light traps (John W. Hock Company, Gainesville, FL, USA) installed from 5 p.m. to 8 a.m. A total of 40 trap-nights were set, with 19 in caves and 21 in open-air settings.

Traps were placed in four separate types of settings: (1) outside of caves (exterior), often in natural forested areas, (2) within the entrance zone of caves, with varying shade and associated fluctuating temperatures, 5–20 m away from the physical opening, depending on the rocky structure, (3) within the dark zone (interior) of caves, completely removed from sunlight and with generally constant temperatures, at a distance less than 100 m from the cave entrance, and (4) far into the caves at a distance larger than 100 m from the cave entrance, often at the end of the cave passage.

The localities of all of the Phlebotomine sandflies captured during the May 2003 field trip include (according to WGS84 system):

- Camp of Andrafiabe (S12.93111° E49.05622°; altitude: 25 m above sea level),
- Andrafiabe cave (S12.93006° E49.05936°; altitude: 25 m a.s.l.),
- Antsirohandoa Skeleton cave (S12.93611° E49.05650°; altitude: 50 m a.s.l.), and
- Camp of Anilotra (S12.93611° E49.05650°; altitude: 125 m a.s.l.).

### Morphological analysis

Sandflies were stored in 96% ethanol. The head, thorax and genitalia were cut off in a drop of ethanol. Soft tissues were lyzed in a bath of KOH 10%, then bleached in Marc-André solution, and mounted between a microscope slide and cover slip in Euparal^®^ for species identification after dehydration in successive alcohol baths. To allow the use of old mounted specimens in chloral gum, some slides were left for several days in a wet room then remounted in Euparal^®^ after complete processing of washing, bleaching, and dehydration. The abdomen related to the specimen was dried and stored in a vial at −20 °C before DNA extraction. Visual analysis of the specimens was performed by means of a BX61 microscope (Olympus, Japan). Measurements and counts were made by using Stream Motion software (Olympus, Japan) and a video camera connected to the microscope.

For both sexes, the following head measurements were made: flagellomeres 1–3; labrum-epipharynx; length of palpal segments 1–5; number of cibarial teeth and denticles (vertical teeth). For males belonging to new species, we also measured the lengths of the sperm pump, aedeagal duct, parameral sheath, epandrial lobe, gonocoxite, gonostyle, and the distance between the accessory spine and the top of the gonostyle. The number of ventral setae of the gonocoxite was also determined.

Drawings were made using a *camera lucida*. Identifications were carried out using the keys and papers [7–12, 26, 30, 32, 33] related to Malagasy Phlebotomine sandflies, as well as other keys [1, 2, 29].

The terminology adopted for the characters is the most recent one for Phlebotomine sandflies [15].

All morphological measurements were tested for normality by a D’Agostino-Pearson omnibus K2 non-parametric normality test (GraphPad Prism version 6.07 for Windows, GraphPad Software, San Diego, CA, USA). Multifactorial analysis (principal component analysis and hierarchical clustering) was also used to investigate the distribution (homogeneity and clustering) of sandfly measurements by means of XLStat 2018 software (Addinsoft, Paris, France).

### Molecular analysis

Genomic DNA was extracted from the first segments of the abdomen of individual sandflies using the QIAmp DNA Mini Kit (Qiagen, Germany) following the manufacturer’s instructions, except for crushing sandfly tissues with a piston pellet (Treff, Switzerland), and using an elution volume of 50–200 μL [7].

A fragment of cytochrome b (Cyt b) (using the C3B-PDR: 5′-CAYATTCAACCWGAATGATA-3′ and N1N-PDR: 5′-GGTAYWTTGCCTCGAWTTCGWTATGA-3′ primers) and the D1 and D2 fragments of 28S rDNA (using the C1′: 5′-ACCCGCTGAATTTAAGCAT-3′ and D2: 5′-TCCGTGTTTCAAGACGGG-3′ primers) were amplified by PCR [13, 14].

Amplicons were analyzed by electrophoresis in 1.5% agarose gel containing Gel Green at a concentration of 0.005% V/V. Direct sequencing in both directions was performed using the primers used for DNA amplification. Sequence correction was performed by using Pregap and Gap softwares included in the Staden Package [5].

Consensus sequences were aligned by the Clustal W algorithm [38] from the BioEdit 4.8.10 sequence editor [19], and corrected manually.

The sequences obtained during the present study have been compared with all the sequences available in GenBank related to the cyt b and D1-D2 sequences of sandflies from Madagascar and the Comoros Archipelago.

Sequence data were analyzed by MEGA7 [24] based on maximum likelihood. The maximum likelihood trees were constructed by using the substitution model HKY85. All positions containing gaps and missing data were removed from the analyses.

## Results

A total 723 Phlebotomine sandflies were used for morphological and/or molecular analyses (Table 1).

**Table 1:** Distribution of sandflies collected in the Ankarana *tsingy.*

Genus	Subgenus	Species	Females	Males	Total	Species (%)	Subgenus (%)	Genus (%)
*Phlebotomus*	*Madaphlebotomus*	*fertei*	38	82	120	16.60	16.60	16.60
*Sergentomyia*	*Vattieromyia*	*sclerosiphon*	218	150	368	50.90	75.93	82.85
		*anka*	97	84	181	25.03		
	*Rondanomyia*	*goodmani*	1		1	0.14	0.69	
		*ozbeli* n. sp.	4		4	0.55		
	*Ranavalonomyia* subg. nov.	*volfi* n. sp.	16	13	29	4.01	4.01	
	*Riouxomyia* subg. nov.	*kaltenbachi* n. sp.	1		1	0.14	0.14	
	Undetermined				15	2.07	2.07	
*Grassomyia*		spp	1	3	4	0.55	0.55	0.55
Total			376	332	723	100	100	100

The sandflies belonged to three genera: *Phlebotomus*, *Sergentomyia*, and *Grassomyia*, in accordance with the systematic revision of Abonnenc & Léger [3] and considering *Grassomyia* as a genus and not as a subgenus of *Sergentomyia*. Our sampling included at least nine species (Table 2).

**Table 2:** Summary of sandflies collection in the Ankarana *tsingy* in relation with trap position.

	Location of traps				Total
	Outside	Entrance of the cave	Middle cave	Cave bottom	
Number of traps set up	21	4	10	5	40
Number of positive traps	17	4	6	1	28
Number of sandflies caught	248	127	344	5	724
Mean number of sandflies per trap	11.8	31.8	34.4	1.0	18.1
*Ph. fertei*	19	71	30	0	120
*Se. anka*	99	12	70	0	181
*Se. sclerosiphon*	102	42	221	3	368
*Se. goodmani*	1	0	0	0	1
*Se. ozbeli* n. sp.	1	1	2	0	4
*Se. volfi* n. sp.	15	0	12	2	29
*Se. kaltenbachi* n. sp.	0	0	1	0	1
*Grassomyia* spp.	4	0	0	0	4
Undetermined	8	1	6	0	15

In the Ankarana collection, only one species in the genus *Phlebotomus*, *Ph. fertei*, was identified and was represented by 120 specimens. The subgenus *Vattieromyia* of the genus *Sergentomyia* was the most abundant, with *Se. sclerosiphon* representing about 50% of the sandflies caught and *Se. anka* about 25%.

With regards to the genus *Grassomyia*, only four specimens (three males and one female) were captured. Taking into account: (i) our limited sampling, (ii) the process involved for molecular analysis of the thorax, and (iii) the unresolved systematics of this group, we limited our identifications to the genus level. The female exhibited 40 cibarial teeth, which is in agreement with *Gr. madagascariensis* according to Abonnenc (1969). The males, however, exhibited 12, 19, and 25 cibarial teeth, which is more than the 10–15 teeth reported by Abonnenc (1969) for this species [1]. Hence, our samples probably included more than one species.

Regarding the genus *Sergentomyia*, one specimen of *Se. goodmani* was captured. This species was previously only known from the Namoroka *tsingy* and Ankiliefatra.

Three undescribed species of *Sergentomyia* were collected at Ankarana. According to the morphology of their spermathecae, two of these taxa could not be classified into any existing subgenus. We thus propose two new subgenera.

The number of individuals collected was notably variable, depending on the location of the traps (Table 2). Four trap positions were used with respect to the cave sites: outside, in the entrance, in the middle section, and deep inside. Interesting differences were noted concerning the number of sandflies caught per trap. The mean number of sandflies per trap was the highest in the middle section and in the cave entrance. A significantly lower number was observed outside, and the lowest mean number of captures was observed deep inside the caves (one sandfly recorded per trap). *Phlebotomus fertei*, *Se. anka*, *Se. sclerosiphon*, *Se. volfi* n. sp., and *Se. ozbeli* n. sp. were captured inside as well as outside of caves. *Grassomyia* spp. were not found in caves, which is in agreement with the biology of this genus [2]. The single specimen of *Se. goodmani* was obtained outside caves, whereas the single specimen of *Se. kaltenbachi* n. sp. was from within caves.

All sequences analyzed in the present study have been deposited in GenBank under the accession numbers MK465124–MK465180 and MK465182 (cyt B) and MK452276–MK452286 and MK452288–MK452340 (D1-D2 rDNA sequences), except for some specimens which could not be amplified or sequenced.

**Figure 3:**
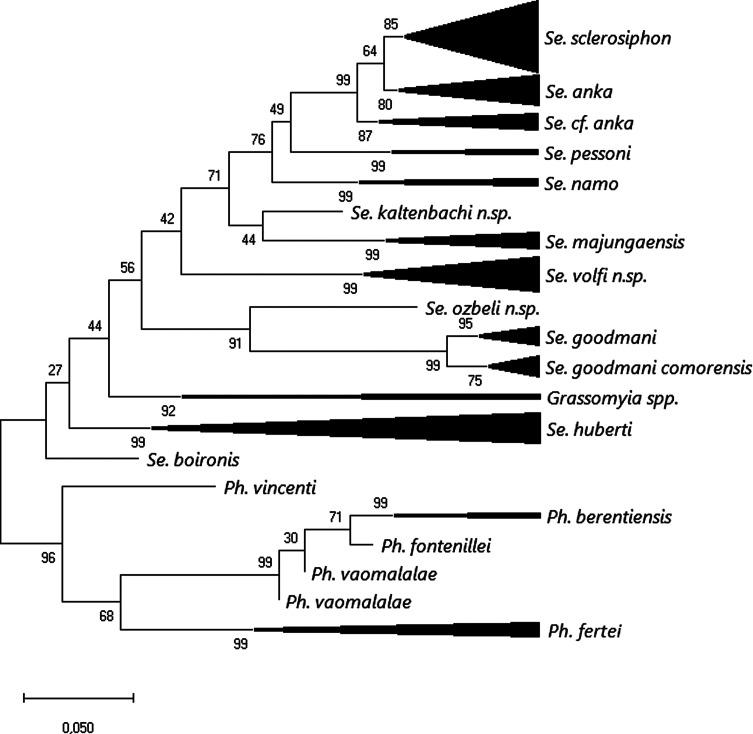
Maximum likelihood tree obtained from partial cyt B sequences based on the HKY85 model after rooting on *Phlebotomus* spp. The tree with the highest log likelihood (−2203.9) is shown. The percentage of trees in which the associated taxa clustered together is shown next to the branches. The tree is drawn to scale, with branch lengths measured in the number of substitutions per site. The analysis involved 126 nucleotide sequences.

After rooting on available *Madaphlebotomus* sequences, maximum likelihood analysis carried out on a 620 bp database of cyt B sequences (Fig. 3) showed strong individualization of all species, except for *Vattieromyia*, and most of the specimens were grouped according to their morphological identification. Some specimens, however, doubtfully identified by morphology as *Se. cf anka* (MADA96, 199, 819, 876, 897, 898 and 1345) were branched together. *Sergentomyia kaltenbachi* n. sp. was included in the *Vattieromyia* branch. Regarding the subgenus *Rondanomyia*, our specimen of *Se. goodmani* was grouped with sequences from this species deposited in GenBank and from Ankiliefatra and Namoroka. Its distribution now covers the North, the Centre and the south of Madagascar. The taxon *Se. ozbeli* n. sp. is the sister species of [*Se. goodmani* + *Se. goodmani comorensis*]. All the *Sergentomyia volfi* n. sp. specimens were strongly grouped together with high intraspecific variability of 1.6% (Table 3). Lastly, the genetic distance between the two sequenced *Grassomyia* specimens of more than 10% clearly suggests that they do not belong to the same species.

**Table 3:** Genetic distances between taxa based on cyt B mtDNA sequences. In bold, the intraspecific distances.

	*Se. sclerosiphon*	*Se. anka*	*Se. cf anka*	*Se. pessoni*	*Se. namo*	*Se. kaltenbachi* n. sp.	*Se. majungaensis*	*Se. ozbeli* n. sp.	*Se. goodmani*	*Se. goodmani comorensis*	*Se. volfi* n. sp.	*Se. boironis*	*Se. huberti*	*Grassomyia spp.*	*Ph. vincenti*	*Ph. berentiensis*	*Ph. fontenillei*	*Ph. vaomalalae*	*Ph. fertei*
*Se. sclerosiphon*	**0.003**																		
*Se. anka*	0.028	**0.005**																	
*Se. cf anka*	0.045	0.047	**0.025**																
*Se. pessoni*	0.089	0.091	0.098	**0.006**															
*Se. namo*	0.077	0.086	0.088	0.093	**0.001**														
*Se. kaltenbachi* n. sp.	0.124	0.123	0.13	0.122	0.122	**–**													
*Se. majungaensis*	0.097	0.113	0.108	0.112	0.106	0.124	**0.004**												
*Se. ozbeli* n. sp.	0.156	0.166	0.162	0.168	0.153	0.164	0.152	**–**											
*Se. goodmani*	0.178	0.177	0.176	0.18	0.169	0.175	0.184	0.149	**0.006**										
*Se. goodmani comorensis*	0.179	0.173	0.178	0.182	0.176	0.176	0.178	0.154	0.153	**0.018**									
*Se. volfi* n. sp.	0.153	0.157	0.158	0.141	0.138	0.163	0.139	0.162	0.192	0.193	**0.015**								
*Se. boironis*	0.118	0.122	0.124	0.129	0.105	0.16	0.12	0.157	0.164	0.168	0.134	**–**							
*Se. huberti*	0.145	0.144	0.155	0.15	0.139	0.16	0.138	0.164	0.177	0.176	0.155	0.091	**0.033**						
*Grassomyia* spp.	0.159	0.155	0.162	0.156	0.144	0.174	0.156	0.179	0.19	0.191	0.155	0.131	0.136	**0.1**					
*Ph. vincenti*	0.173	0.175	0.184	0.176	0.172	0.193	0.161	0.198	0.17	0.178	0.199	0.132	0.165	0.178	**–**				
*Ph. berentiensis*	0.19	0.181	0.19	0.192	0.182	0.212	0.186	0.23	0.212	0.214	0.199	0.153	0.169	0.162	0.148	**0**			
*Ph. fontenillei*	0.188	0.183	0.195	0.195	0.18	0.214	175	0.24	0.213	0.219	0.212	0.151	0.164	0.16	0.144	0.032	**–**		
*Ph. vaomalalae*	0.182	0.178	0.184	0.191	0.17	0.203	0.158	0.224	0.202	0.198	0.2	0.142	0.161	0.159	0.148	0.043	0.032	**0.012**	
*Ph. fertei*	0.173	0.169	0.18	0.168	0.189	0.214	0.175	0.213	0.216	0.217	0.198	0.144	0.16	0.168	0.14	0.145	0.144	0.135	**0.006**

**Figure 4:**
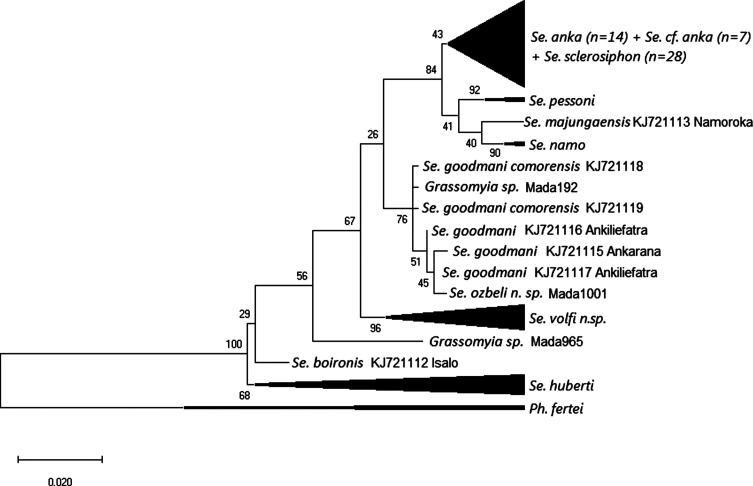
Maximum likelihood tree obtained from partial 28S rDNA sequences (D1 and D2 domains) based on the HKY85 model after rooting on *Phlebotomus fertei*. The tree with the highest log likelihood (−980.66) is shown. The percentage of trees in which the associated taxa clustered together is shown next to the branches. The tree is drawn to scale, with branch lengths measured in the number of substitutions per site. The analysis involved 126 nucleotide sequences.

Maximum likelihood analysis carried out on a 520 bp database of D1-D2 rDNA sequences (Fig. 4) showed little variability between most of the specimens referred to as both *Se. sclerosiphon* and *Se. anka*, with the exception of specimens labelled MADA95, 199, 876, 893, 897, and 1345, which were on a separate branch. *Sergentomyia majungaensis*, an ungrouped *Sergentomyia*, was included in the *Vattieromyia* clade.

Only one *Grassomyia* specimen was well isolated.

Within the *Rondanomyia* subgenus, some variability was noted between *Se. goodmani* and *Se. goodmani comorensis*. The six variable nucleotide positions could explain the mixing of the both taxa. *Sergentomyia ozbeli* n. sp. was mixed with these taxa.

All specimens morphologically identified as *Se. volfi* n. sp. were included in a well-defined clade.

Genetic distances calculated on rDNA sequences between and within taxa are provided in Table 4.

**Table 4:** Genetic distances between taxa based on D1 and D2 rDNA sequences. In bold, the intraspecific distances.

	*Se. sclerosiphon*	*Se. anka*	*Se. cf anka*	*Se. pessoni*	*Se. namo*	*Se. majungaensis*	*Se. goodmani*	*Se. goodmani comorensis*	*Se. ozbeli* n. sp.	*Se. volfi* n. sp.	*Se. huberti*	*Se. boironis*	*Grassomyia*	*Ph. fertei*
*Se. sclerosiphon*	**0.002**													
*Se. anka*	0.004	**0.001**												
*Se. cf anka*	0.007	0.006	**0.003**											
*Se. pessoni*	0.007	0.009	0.01	**0**										
*Se. namo*	0.011	0.011	0.009	0.012	**0**									
*Se. majungaensis*	0.016	0.018	0.016	0.019	0.014	**–**								
*Se. goodmani*	0.025	0.027	0.029	0.029	0.027	0.026	**0.003**							
*Se. goodmani comorensis*	0.021	0.023	0.026	0.026	0.022	0.027	0.006	**0.003**						
*Se. ozbeli* n. sp.	0.026	0.027	0.032	0.027	0.028	0.025	0.005	0.008	**–**					
*Se. volfi* n. sp.	0.02	0.022	0.023	0.022	0.025	0.024	0.017	0.017	0.018	**0**				
*Se. huberti*	0.027	0.03	0.031	0.03	0.028	0.033	0.031	0.031	0.031	0.028	**0.002**			
*Se. boironis*	0.033	0.036	0.037	0.034	0.034	0.036	0.032	0.032	0.034	0.031	0.012	**–**		
*Grassomyia* spp	0.034	0.035	0.037	0.036	0.035	0.034	0.021	0.019	0.022	0.027	0.034	0.037	**0.037**	
*Ph. fertei*	0.109	0.11	0.113	0.106	0.104	0.111	0.101	0.104	0.101	0.112	0.095	0.101	0.105	**0**

The analysis of cytochrome b sequences enabled us to highlight a clade close to that of *Sergentomyia anka*. Morphometric analysis of the sandflies involved (31 females) by means of multifactorial approaches (principal component analysis and hierarchical clustering) was then carried out. Twelve head parameters were investigated: flagellomeres 1–3 (AIII, AIV, and AV), labrum (Lb), maxillary palpal segments (P1–P5), and first, second, and third row of vertical teeth (D1R, D2R, and D3R).

**Figure 5:**
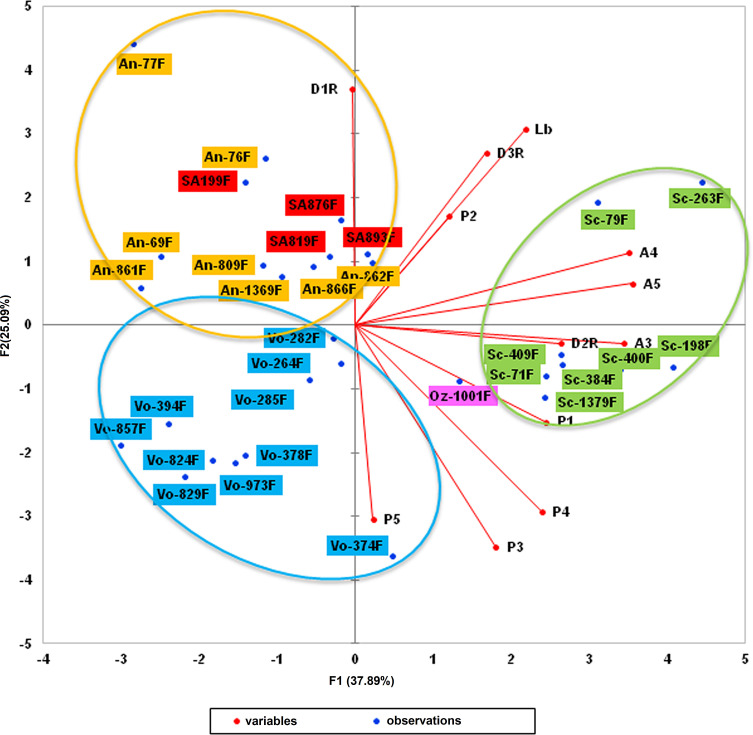
First factorial plan (F1 and F2 axes). Sc = *Se. sclerosiphon*; An = *Se. anka*; SA = *Se.* cf. *anka*; Vo = *Se. volfi* n. sp.; Oz = *Se. ozbeli* n. sp. (all specimens = ♀).

**Figure 6:**
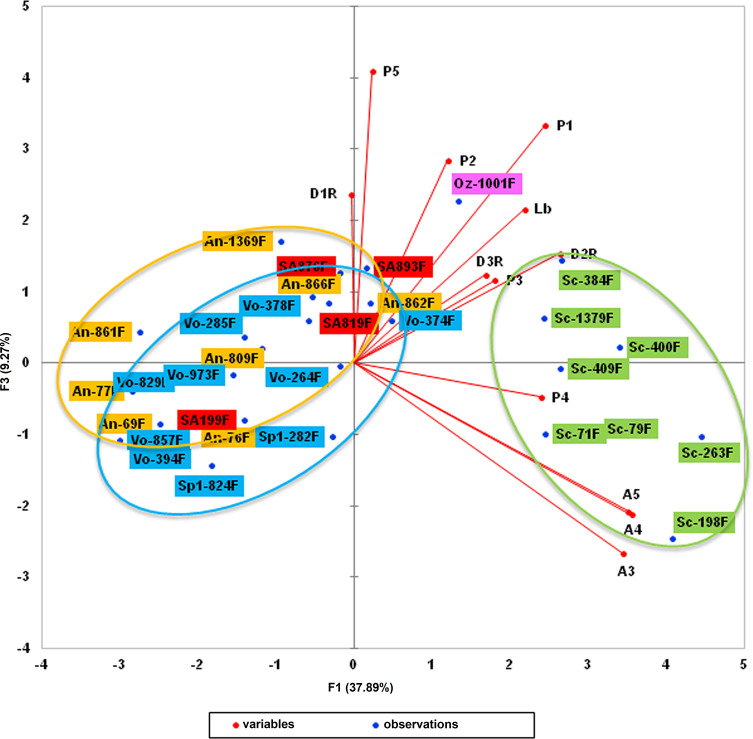
Second factorial plan (F1 and F3 axes). Sc = *Se. sclerosiphon*; An = *Se. anka*; SA = *Se.* cf. *anka*; Vo = *Se. volfi* n. sp.; Oz = *Se. ozbeli* n. sp. (all specimens = ♀).

**Figure 7:**
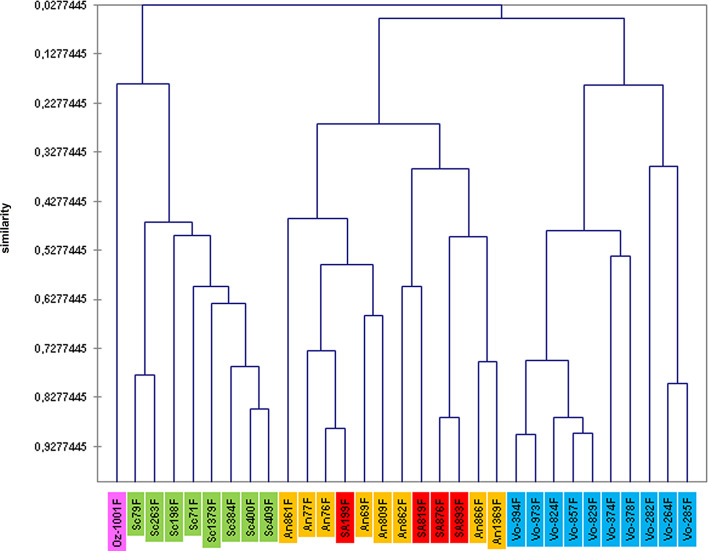
Hierarchical clustering based on morphometrical characters of females belonging to the following species Sc = *Se. sclerosiphon*; An = *Se. anka*; SA = *Se.* cf. *anka*; Vo = *Se. volfi* n. sp.; Oz = *Se. ozbeli* n. sp.

Principal component analysis yielded a variability of more than 37% for the F1 axis, 25% for F2, and 9% for F3 (Table 5). Parameters with the highest contribution (more than 5%) for the F1 axis were flagellomeres AIII, AIV, and AV, palpal segments P1 and P4, and the second row of vertical teeth (D2R). Labrum (Lb) represented 13.9% of the variability along the F2 axis, along with palpal segments (P3–P5), and the first and third row of vertical teeth (D1R and D3R) (Table 6). Overall, the different parameters investigated accounted for more than 81% of the variability of the F1 axis and more than 89% of the variability of the F2 axis. Figures 5–7 display the clustering of the 31 sandflies studied.

**Table 5:** Principal component analysis results (raw values).

	F1	F2	F3	F4	F5	F6	F7	F8	F9	F10	F11	F12
Raw value	4.547	3.011	1.112	0.883	0.728	0.563	0.440	0.342	0.193	0.143	0.030	0.008
Variability (%)	37.891	25.090	9.266	7.355	6.066	4.695	3.666	2.852	1.608	1.188	0.254	0.069
Cumulative	37.891	62.981	72.248	79.602	85.668	90.363	94.029	96.880	98.488	99.677	99.931	100

**Table 6:** Parameter contribution (%) to the total variability (highest contributions displayed in bold).

Variable	F1	F2	F3
A3	**17.549**	0.125	**10.606**
A4	**18.232**	1.890	6.423
A5	**18.622**	0.620	6.682
Lb	7.008	**13.950**	6.880
P1	**8.791**	3.409	**16.410**
P2	2.125	4.287	**11.928**
P3	4.771	**17.996**	1.985
P4	**8.424**	**12.633**	0.341
P5	0.075	**13.821**	**24.702**
D1R	0.003	**20.343**	**8.305**
D2R	**10.218**	0.120	3.491
D3R	4.183	**10.807**	2.248
	81.8%	89.6%	72%

Hierarchical clustering analysis was able to find slightly more homogeneous groups of *Sergentomyia* (Fig. 7). It also confirmed that the four sandfly specimens which formed a clade belong to the *Se. anka* species.

### *Sergentomyia (Ranavalonomyia) volfi* Depaquit, Blavier & Laroche n. sp.

urn:lsid:zoobank.org:act:80C255F3-9A66-4A10-880A-6933641FBFBB

Genus: *Sergentomyia* França & Parrot 1920

Subgenus: *Ranavalonomyia* subg. nov. Depaquit, Blavier & Laroche (type-species: *Se. volfi* n. sp.)

Authorship: note that the authors of the new taxon are different from the authors of this paper; Article 50.1 and Recommendation 50A of International Code of Zoological Nomenclature [21].

Etymology: the species is dedicated to our Czech colleague Petr Volf.

Type-locality: *tsingy* of Ankarana, Madagascar.

Type-specimens: holotype female (MADA1011) and five paratypes (two males labelled MADA825 and MADA899 and three females labelled MADA829, MADA895 and MADA901) deposited in the *Muséum National d’Histoire Naturelle*, Paris, France (MNHN).

#### Female (Figures 8 and 9)

A total of 16 specimens were examined. Counts and measurements indicated in the description are those of the holotype MADA1011.

**Figure 8:**
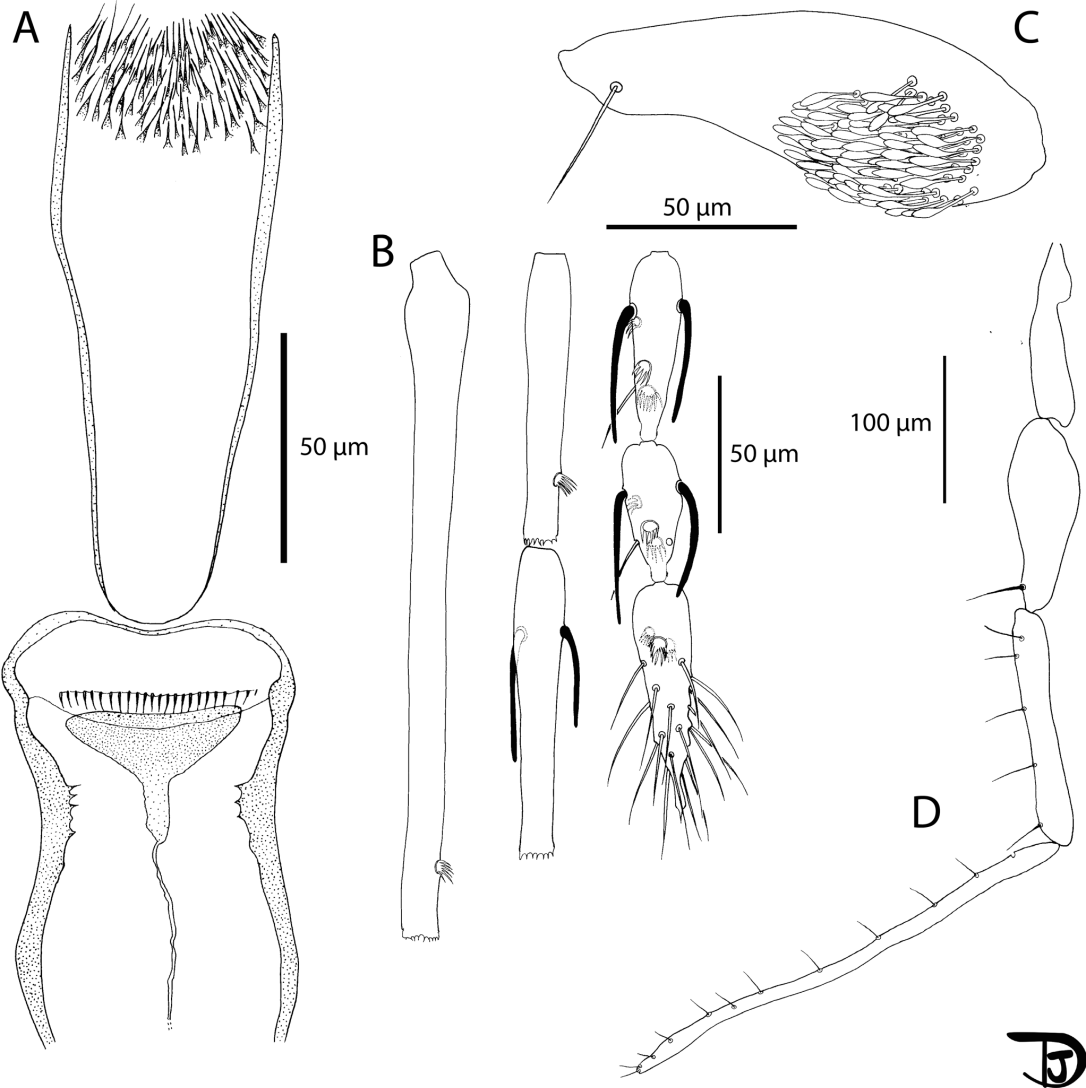
*Sergentomyia (Ranavalonomyia) volfi* n. sp. female holotype (MADA1011). (A) pharynx and cibarium, (B) flagellomeres 1, 2, 3, 12, 13 & 14, (C) third palpal segment, (D) palp.

**Figure 9:**
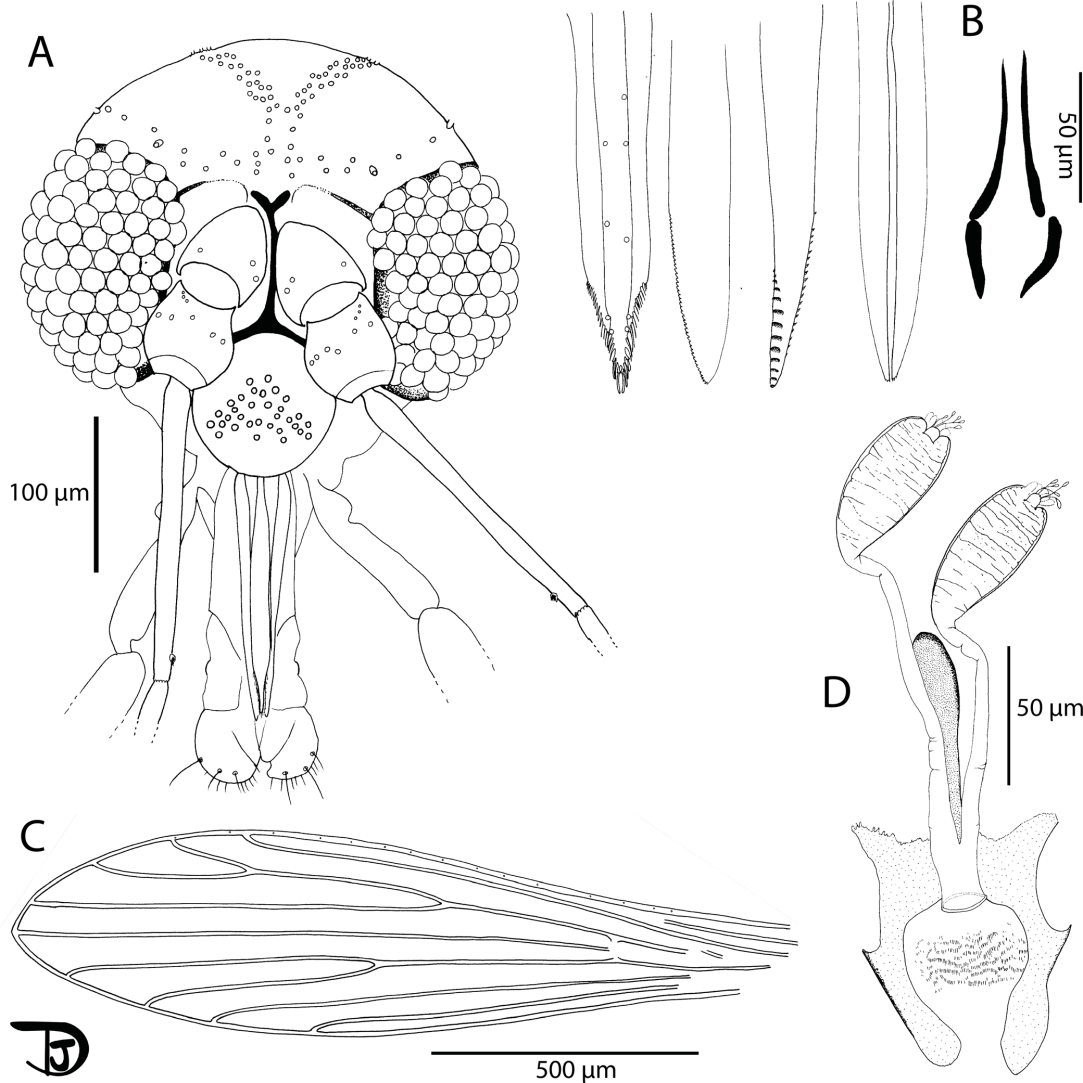
*Sergentomyia (Ranavalonomyia) volfi* n. sp. female. (A) head (paratype MADA895), (B) mouth parts: labrum-epipharynx, mandible, maxilla, hypopharynx & labial furca, respectively (paratype MADA901), (C) wing (paratype MADA829), (D) spermathecae, not smooth, showing wrinkles, and genital furca (paratype MADA901).

##### Head

Occiput with two narrow lines of well individualized setae. On the line above the eyes, two greater insertions of setae on each side. Clypeus 81 μm long, 63 μm wide, with about 30 setae randomly distributed. Eyes 188/107 μm with about 100 facets.

Interantennal suture incomplete. Interocular suture not reaching the interantennal one.

Flagellomeres. Flagellomere 1 longer than f2 + f3. Absence of ascoid on f1 and f2. Ascoidal formula: 2/f3 − f13. One distal papilla on f1 and f2. Absence of papilla from f3 to f11. Three papillae on flagellomeres 12–14. Absence of simple setae on flagellomeres f1–f3. One simple seta on flagellomeres f4–f8, f10, and f12. Two simple setae on f9, f11, and f13. About 20 simple setae on f14. Palpi showing a very wide P3. Palpal formula: 1–5. Presence of many Newstead’s sensilla on P3 (74 on the holotype) implanted basally. Absence of Newstead’s sensilla on P2. Presence of one distal spiniform seta on P3 and of five spiniform setae on P4.

Labrum-epipharynx 187 μm long. Hypopharynx with three small apical teeth on each side of the salivary canal. Maxillary lacinia with about 10 large distal external teeth and about 20 internal ones. Labium showing an open labial suture. Cibarium with 24 clear and palissadic teeth organized along a horizontal line. There are about ten dot-like vertical teeth in front, irregularly disposed along a horizontal line. The pigment patch is pale, triangular with an anterior expansion giving it an overall aspect of russula. Well-armed pharynx on its posterior quarter with long pigmented teeth oriented posteriorly and towards the center.

##### Cervix

Presence of two cervical and two ventro-cervical sensilla on each side.

##### Thorax

Length: 556 μm, sclerites medium brown, absence of post-alar setae, absence of paratergital setae, absence of proepimeral setae, absence of upper anepisternal setae, absence of lower anepisternal setae, absence of anepimeral setae, absence of metepisternal setae, absence of metepimeral setae, presence of setae on the anterior region of the katepisternum. Absence of suture between metepisternum and katepimeron. Metafurca mounted laterally; it is not possible to see clearly whether the vertical arms are joined by a membrane or not.

##### Wing

Length: 1930 μm; width: 510 μm. R5 = 1340 μm; alpha (=R2) = 356 μm; beta (=R2 + 3) = 390 μm; gamma (=R2 + 3 + 4) = 327 μm; delta = 349 μm; pi = 142 μm; epsilon (=R3) = 500 μm; theta (=R4) = 998 μm.

##### Legs

Anterior leg: coxa = 284 μm; femur = 691 μm; tibia = 813 μm; tarsomere i = 455 μm; sum of tii, tiii, tiv, tv = 592 μm. Median leg: coxa = 277 μm; femur = 696 μm; tibia = 949 μm; tarsomere i = 537 μm; other tarsomeres broken. Posterior leg: coxa = 305 μm; femur = 765 μm; tibia = 1099 μm; tarsomere i = 626 μm; sum of tii, tiii, tiv, tv = 720 μm. Absence of spines on the metafemur. One verticil of two spines in the middle and one distal spine of the metatarsomere iii was observed on the paratype MADA1010.

##### Abdomen

Setae randomly distributed on tergites ii–v. Tergite VIII with 14 setae on each side.

Tergite IX without protuberance. Cerci = 155 μm. Setae not observed on sternite X.

##### Genitalia

The appearance of the spermathecae in the Marc-André liquid is very different after dehydration for their mounting in Euparal^®^. In the Marc-André liquid, spermathecae appear elongated, with rather thick walls, and are not sclerotized. The body is unsegmented but covered with many transverse parallel folds giving it a striated appearance. The terminal knob, which carries four and five canaliculi terminates in a bulge, is slightly embedded in the distal part of the body with a cloud-like frill around it. After mounting in Euparal^®^, the spermatheca systematically retracts to a rectangular shape and folds tend to fade. Individual ducts are thin and narrow, smooth and non-sclerotized. They come together to form a short common duct opening at the level of the genital chamber.

The furca has wide and well-developed lateral arms and a stem widening towards the apex. The frame of the genital chamber consists of many small spines arranged more or less regularly and aligned in a few rows.

#### Male (Figures 10 and 11)

A total of 14 specimens have been examined. Counts and measurements indicated in the description are those of the paratype MADA825.

**Figure 10:**
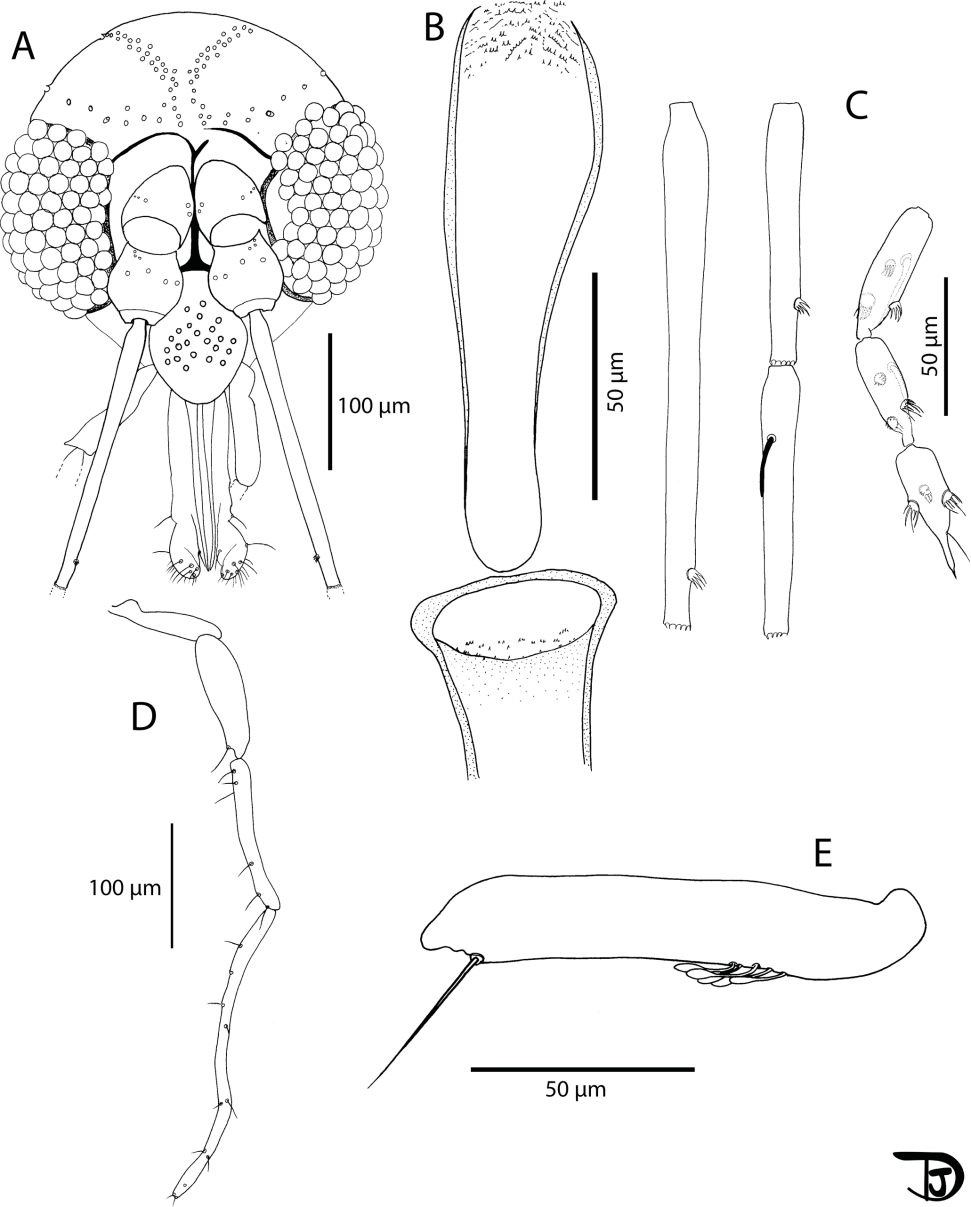
*Sergentomyia (Ranavalonomyia) volfi* n. sp. male. (A) head (paratype MADA825), (B) pharynx and cibarium (paratype MADA825), (C) flagellomeres 1, 2, 3, 12, 13 & 14 (paratype MADA899), (D) third palpal segment (paratype MADA825), (E) palp (paratype MADA899).

**Figure 11:**
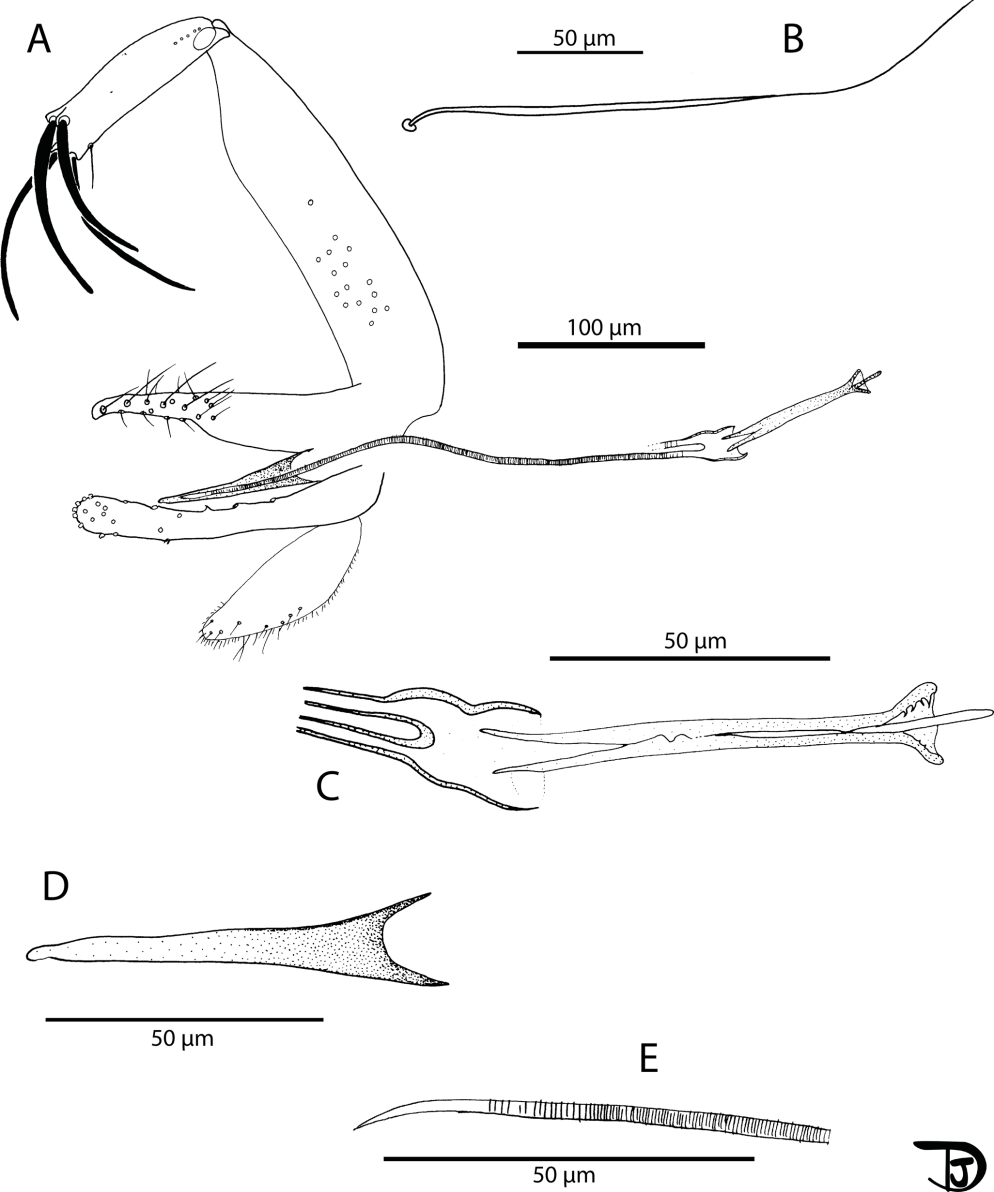
*Sergentomyia (Ranavalonomyia) volfi* n. sp. male (paratype MADA825). (A) genitalia, (B) detail of a ventral coxal seta, (C) sperm pump, (D) parameral sheath, (E) top of the aedeagal ducts.

##### Head

Occiput bearing two well individualized lines of setae. On the infero-lateral line, we can notice two larger insertions on each side. Clypeus bearing 24 setae randomly distributed. Eyes measuring 150 μm high on 85 μm wide, with about 70 facets. Incomplete interocular sutures.

Flagellomeres. Antennal formula: 1/f3 − f13. Absence of ascoid on segments f1 and f2 (=AIII and AIV). One terminal papilla on flagellomeres f1 and f2. One papilla on f11 and three papillae on segments f12–f14. Absence of simple setae on segments f1–f5 as well as f12 and f13. One simple seta from f6 to f8. Two simple setae from f9 to f11 and a dozen on f14. Palpal formula: 1–5. A dozen of Newstead’s sensilla grouped, implanted in the basal quarter of the article. Absence of Newstead’s sensilla on P2. One long spiniform seta distally implanted on P3 and four regularly implanted on P4.

Labrum-epipharynx 140 μm long. Labial suture closed or sometimes very slightly opened. Cibarium armed with ten packs of three to four very small vertical teeth grouped on an irregular line more or less horizontal. In front, some vertical teeth barely observable. Lack of pigment patch. Pharynx armed on its posterior quarter with small pointed teeth oriented backwards, organized along more or less concentric lines.

##### Cervix

Presence of two cervical setae and two ventro-cervical on each side.

##### Thorax

Light brown sclerites, absence of post-alar setae, absence of proepimeral setae, absence of superior or inferior setae on the mesanepisternum, absence of anepimeral setae, absence of setae on the metepisternum, absence of metepimeral setae, presence of setae in the anterior area of the katepisternum. Absence of suture between the metepisternum and the katepimeron. Metafurca mounted laterally; it is not possible to see clearly whether the vertical arms are joined by a membrane or not.

##### Wing

Length: 1280 μm; width: 308 μm. R5 = 931 μm; alpha (=R_2_) = 118 μm; beta (=R_2+3_) = 253 μm; gamma (=R_2+3+4_) = 302 μm; delta = −8 μm; pi = 50 μm; epsilon (=R_3_) = 231 μm; theta (=R_4_) = 602 μm.

##### Legs

Anterior leg: coxa = 256 μm; femur = 520 μm; tibia = 594 μm; tarsomere i = 318 μm; sum of the tarsomeres tii, tiii, tiv and tv = 434 μm. Median leg: coxa = 250 μm; femur = 543 μm; tibia = 734 μm; tarsomere i = 375 μm; sum of the tarsomeres tii, tiii, tiv and tv = 505 μm. Posterior leg: coxa = 270 μm; femur = 580 μm; tibia = 885 μm; tarsomere i = 473 μm; sum of the tarsomeres tii, tiii, tiv and tv = 564 μm. Absence of spines on the metafemur. Two setae implanted in the middle of the metatarsomere iii and one implanted distally.

##### Abdomen

Setae distributed randomly over ii–v. Absence of papilla on the tergites ii–vii.

##### Genitalia

Absence of abdominal sclerotised rods. Gonocoxite carrying a tuft of about twenty internal setae. Absence of basal lobe. Gonostyle bearing four thick, curved terminal spines (or two terminal and two subterminal spines). Accessory spine implanted very distally. Simple paramere, slightly curved at its apex with a vestiture occupying the inner face of the distal half. Parameral sheath straight, finger-like, narrowing gradually from the proximal end to the distal end which is rounded. Striated aedeagal ducts, pointed at their apex. Sperm pump with a long thin piston. Epandrial lobes shorter than the gonocoxite. Cerci = 100 μm.

### *Ranavalonomyia* Depaquit, Blavier & Laroche subg. nov.

urn:lsid:zoobank.org:act:D7CCB6F7-3AF5-43B7-AEAF-CADE0CDC7384

Both sexes: labial suture open; absence of ascoid on flagellomeres f1 and f2 (=AIII and AIV); presence of an apical fringe on the top of f1–f6 and absence of setae on the following sclerites of the thorax: proepimeron, anepisternum, anepimeron, katepimeron, metepisternum and metepimeron. Female: third palpal segment particularly wide, with a large number of basal Newstead’s sensilla; spermathecae slightly wrinkled, without sclerification.

Type-species: *Sergentomyia (Ranavalonomyia) volfi* Depaquit, Blavier & Laroche n. sp.

Etymology: the subgenus is dedicated to Ranavalona III, the last Queen of Madagascar.

### *Sergentomyia (Rondanomyia) ozbeli* Depaquit, Blavier & Randrianambinintsoa n. sp.

urn:lsid:zoobank.org:act:D96B70E2-9B0C-42FA-AC25-7BB2D5DB4C15

Genus: *Sergentomyia* França & Parrot 1920

Subgenus: *Rondanomyia* Theodor, 1958

Authorship: note that the authors of the new taxon are different from the authors of this paper; Article 50.1 and Recommendation 50A of International Code of Zoological Nomenclature [21].

Etymology: the species is dedicated to our Turkish colleague Yusuf Ozbel.

Type-locality: *tsingy* of Ankarana, Madagascar.

Type-specimens: holotype female (MADA1001) and one paratype female (mounted *in toto* on a slide with seven other sandflies) deposited in the *Muséum National d’Histoire Naturelle*, Paris, France (MNHN).

The measurements indicated below are those related to the holotype labelled MADAIT13 except for the legs, measured on the paratype MADA1001.

Number of specimens examined = 3.

#### Female (Figures 12 and 13)

##### Head

Occiput with two narrow lines of setae well individualized. On the line above the eyes, two larger insertions of setae on each side. Clypeus 110 μm long, 184 μm wide, with setae randomly distributed. Eyes 396/210 μm with more than 130 facets. Interantennal suture not complete. Interocular sutures thick.

**Figure 12:**
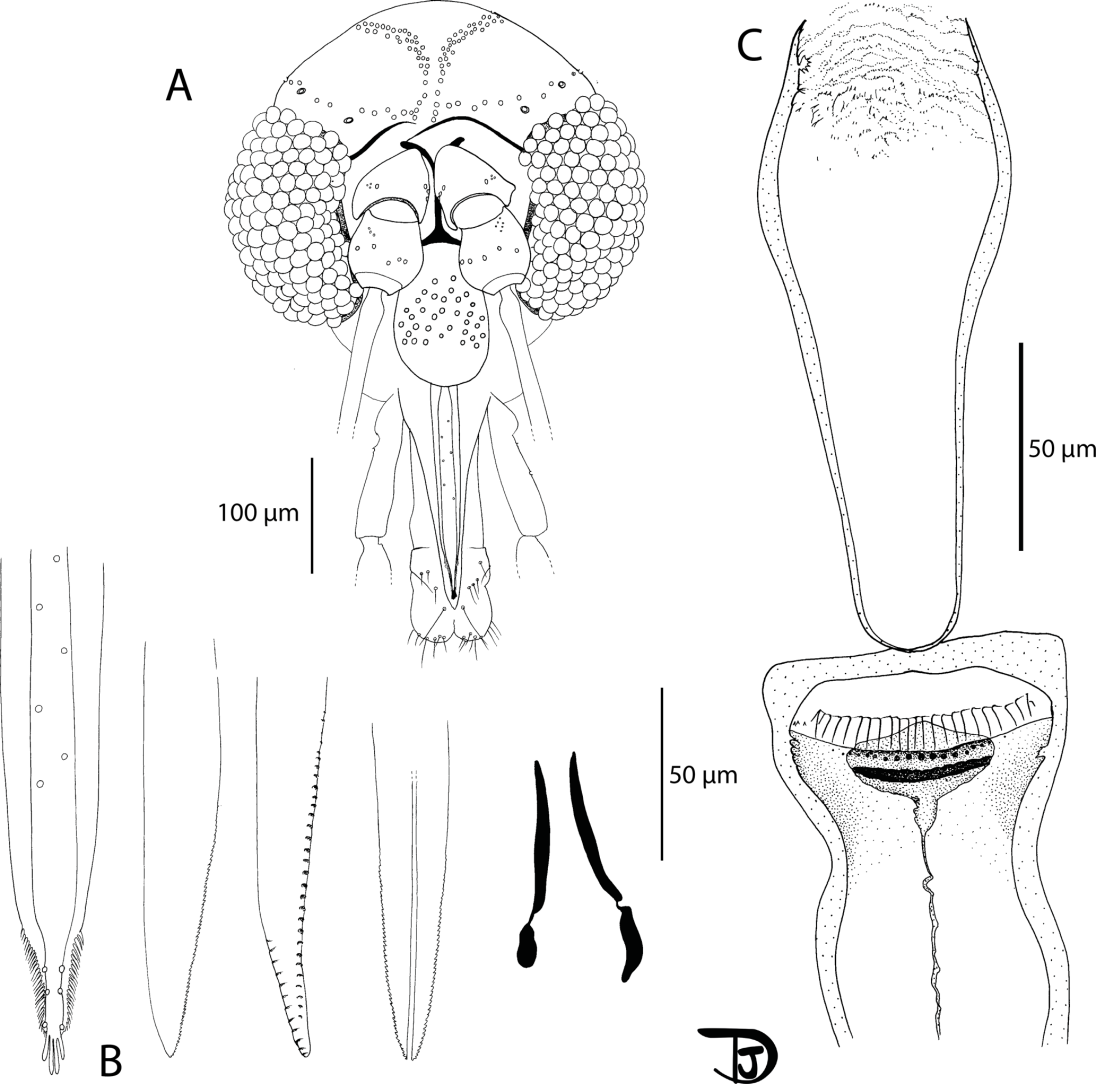
*Sergentomyia (Rondanomyia) ozbeli* n. sp. female (holotype MADA1001). (A) head, (B) mouth parts: labrum-epipharynx, mandible, maxilla, hypopharynx & labial furca, respectively, (C) pharynx and cibarium.

**Figure 13:**
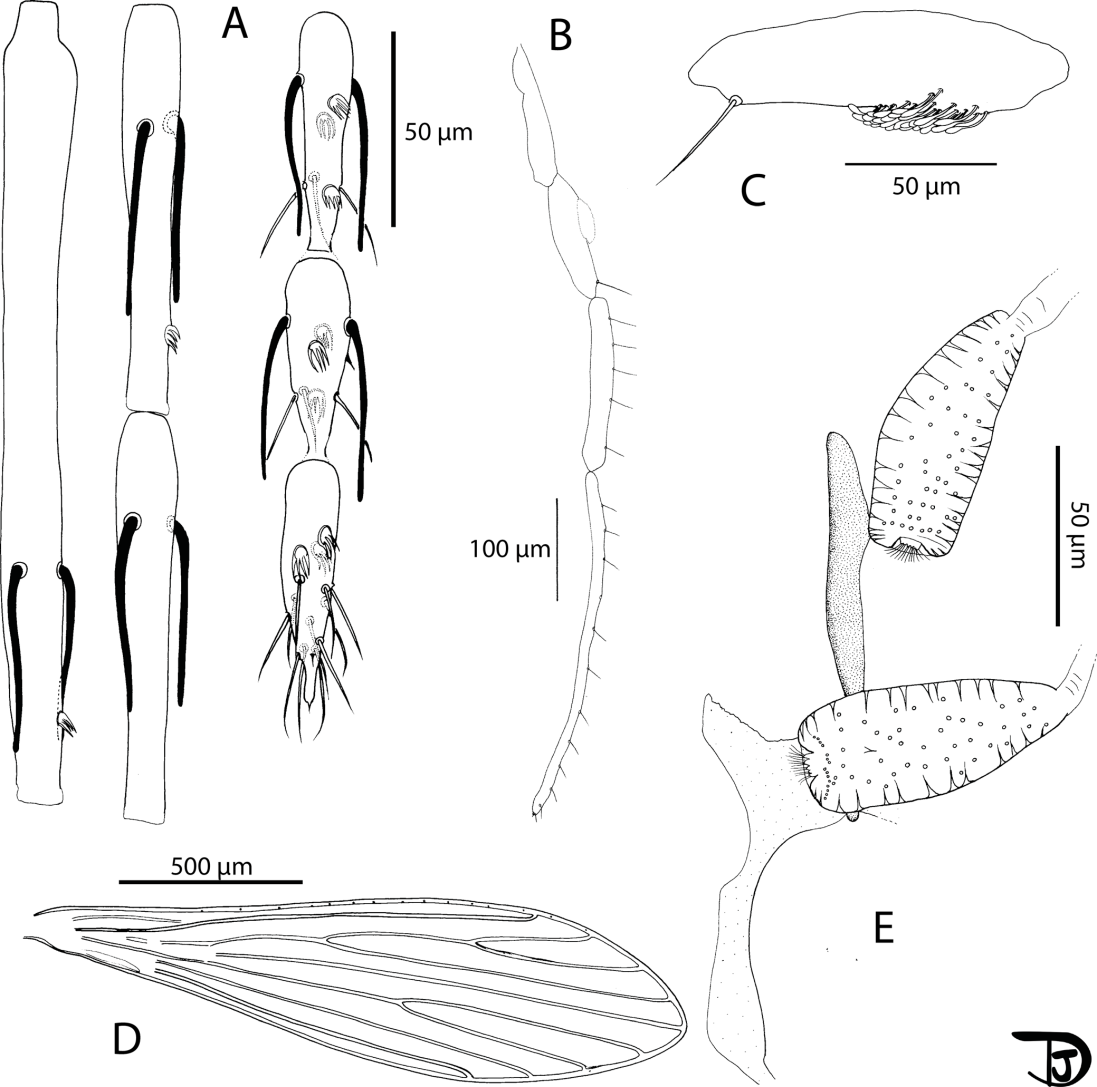
*Sergentomyia (Rondanomyia) ozbeli* n. sp. female (holotype MADA1001). (A) flagellomeres 1, 2, 3, 12, 13, and 14; pharynx and cibarium, (B) palp, (C) third palpal segment, (D) wing, (E) spermathecae and genital furca.

Flagellomere 1 as long as f2 + f3. Flagellomeres exhibiting two ascoids from f1 to f13. The internal and external ascoids on f1 are implanted at the same level; On the holotype, one terminal papilla on f1 and f2; one median papilla on f9–f11. Three papillae on f12–f14. On the holotype, no simple setae have been observed from f1 to f6; two from f7 to f11; three on f12 and f13; about ten simple setae and one spine on f14. Palpal formula: 1–5. About 50 basal Newstead’s sensillae on p3. No Newstead’s sensilla on p2. One distal spiniform seta on p3 and 5 on p4, regularly implanted.

Labrum-epipharynx 197 μm long with lateral spines at the top; Hypopharynx with very small apical teeth. Maxillary lacinia with about 25 internal teeth and 10 external teeth at the top; Labium exhibiting an open labial suture. Cibarium with a line of about 20 colorless posterior teeth. The central ones are more or less identical to the lateral ones, except for a few vertical teeth on each side. One row of anterior vertical teeth of dot-like appearance. The central ones are bigger than the lateral ones. In front of the latter row, there is a dark wavy line, parallel to the lines of teeth, completely traversing the sclerotized area (=pigment patch) exhibiting a posterior expansion.

Pharynx armed with small dot-like teeth arranged along more or less concentric lines.

##### Cervix

Two cervical sensilla on each side. Ventro-cervical sensilla not observed.

##### Thorax

590 μm long, dark sclerites, presence of post-alar setae, absence of proepimeral setae, absence of upper anepisternal setae, absence of lower anepisternal setae, absence of anepimeral setae, absence of metepisternal setae, absence of metepimeral setae, presence of setae on the anterior region of the katepisternum. Absence of suture between metepisternum and katepimeron. Metafurca mounted laterally; it is not possible to see clearly whether the vertical arms are joined by a membrane or not.

##### Wing

Length: 1824 μm; width: 500 μm. R5 = 1292 μm; alpha (=R_2_) = 332 μm; beta (=R_2+3_) = 390 μm; gamma (=R_2+3+4_) = 264 μm; delta = 184 μm; pi = 245 μm; epsilon (=R_3_) = 390 μm; theta (=R_4_) = 984 μm.

##### Legs

Anterior leg: coxa = 282 μm; femur = 690 μm; tibia = 735 μm; tarsomere i = 411 μm; sum of tii, tiii, tiv, tv = 630 μm. Median leg: coxa = 292 μm; femur = 698 μm; tibia = 845 μm; tarsomere i = 495 μm; sum of tii, tiii, tiv, tv = 660 μm. Posterior leg: coxa = 327 μm; femur = 783 μm; tibia = 1054 μm; tarsomere i = 557 μm; sum of tii, tiii, tiv, tv = 713 μm. Absence of spines on the metafemur. One verticil of two spines on the metatarsomere iii.

##### Abdomen

Setae randomly implanted on tergites ii–v. Presence of about 20 setae on tergite VIII. Absence of protuberance on the tergite IX. Setae not observed on the sternite X.

##### Genitalia

Oval spermatheca exhibiting throughout its length many internal spines rosebush-like in lateral view and dot-like in apical or proximal view. Short and flattened terminal knob, invaginated in the spermatheca, bearing about 10 canaliculi. Furca partly hidden but nevertheless showing a long and narrow stem. Genital chamber observed but impossible to draw. Distal part of the individual ducts of the spermathecae without any sclerotization nor striation. Basal part of the spermathecal ducts not observed on the holotype. On one paratype, observation of a common duct. Cerci = 166/69 μm.

### *Sergentomyia (Riouxomyia) kaltenbachi* Depaquit, Blavier & Randrianambinintsoa n. sp.

urn:lsid:zoobank.org:act:B8FE04DD-CF80-4DDC-B9AB-5E12DE64180A

Genus: *Sergentomyia* França & Parrot 1920

Subgenus: *Riouxomyia* subg. nov. Depaquit, Blavier & Randrianambinintsoa (type-species: *Se. kaltenbachi* n. sp.)

Authorship: note that the authors of the new taxon are different from the authors of this paper; Article 50.1 and Recommendation 50A of International Code of Zoological Nomenclature [21].

Etymology: the species is dedicated to our French colleague Matthieu Kaltenbach.

Type-locality: *tsingy* of Ankarana, Madagascar.

Type-specimen: holotype female (MADA728) deposited in the *Muséum National d’Histoire Naturelle*, Paris, France (MNHN).

The measurements indicated below are those related to the holotype. Some organs broken during the catching, storage or mounting process and impossible to observe.

#### Female (Figures 14 and 15)

##### Head

Occiput with two narrow lines of setae well individualized. Clypeus 105 μm long, 80 μm wide, with setae randomly distributed. Eyes 186/86 μm well developed including about 100 facets. Interantennal and interocular suture complete.

**Figure 14:**
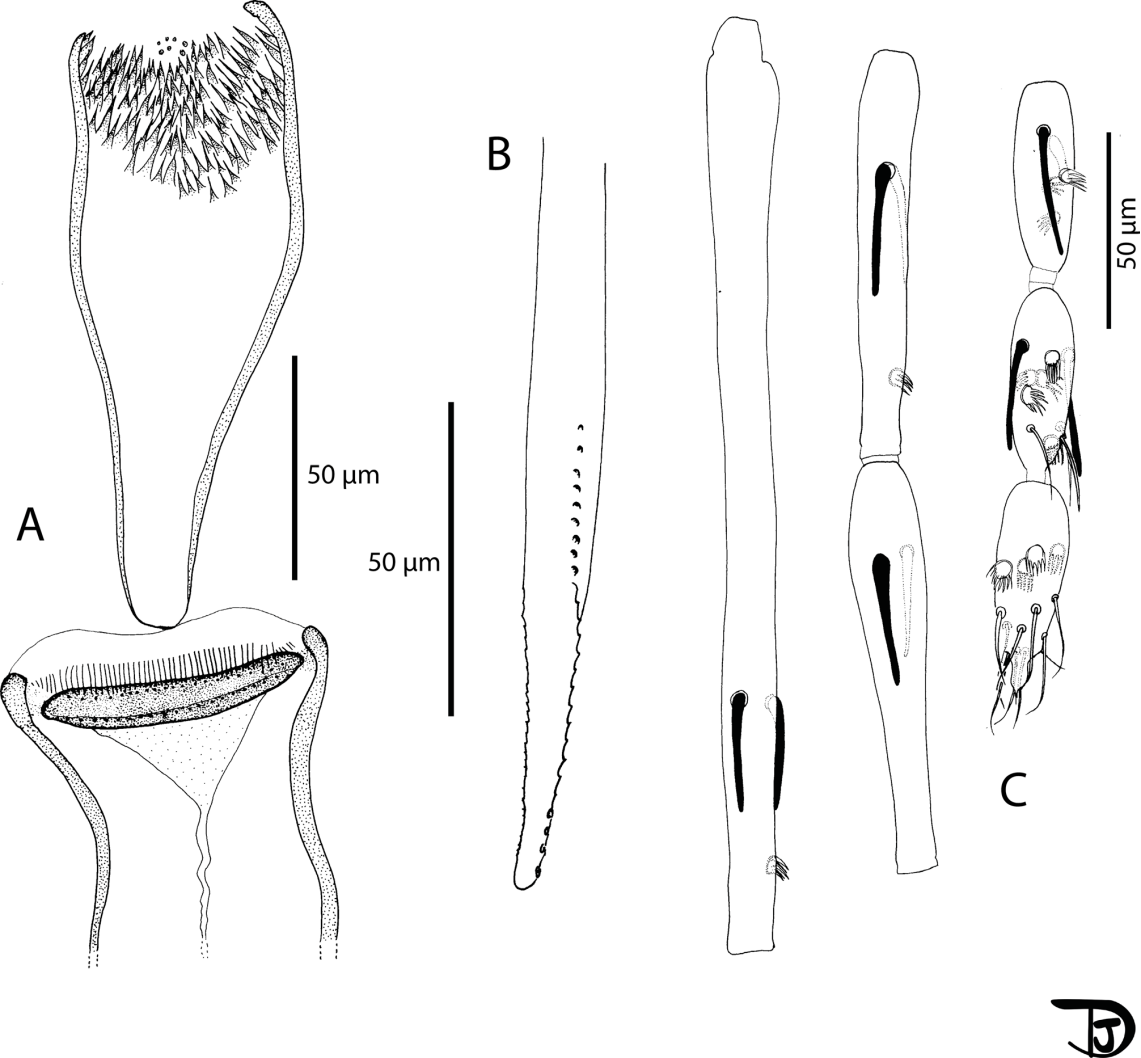
*Sergentomyia (Riouxomyia) kaltenbachi* n. sp. female (holotype MADA728). (A) pharynx and cibarium, (B) maxilla, (C) flagellomeres 1, 2, 3, 12, 13 and 14.

**Figure 15:**
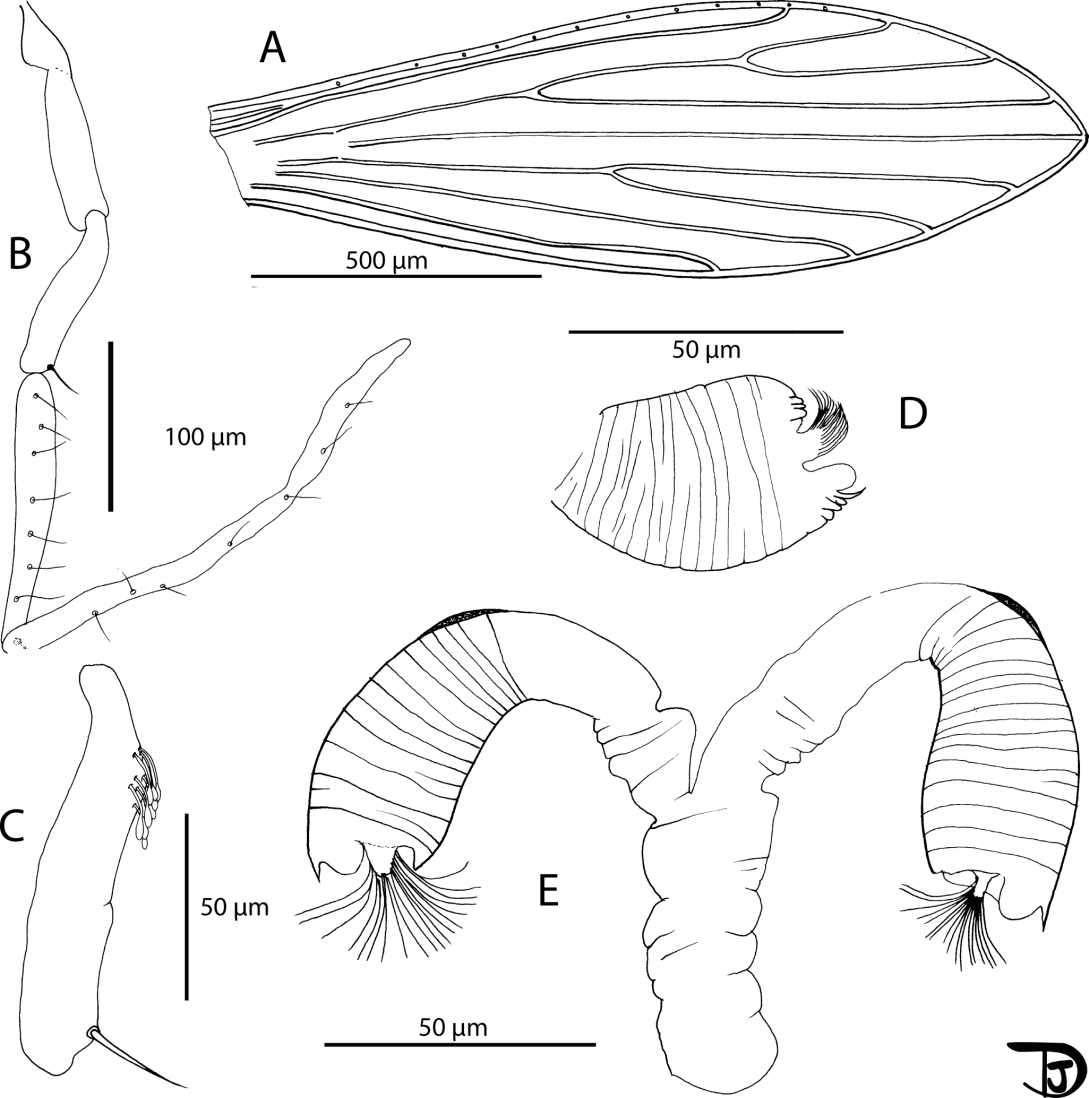
*Sergentomyia (Riouxomyia) kaltenbachi* n. sp. female (holotype MADA728). (A) wing, (B) palp, (C) third palpal segment, (D) spermathecae after mounting, (E) spermathecae drawn in Marc-André.

Flagellomere 1 longer than f2 + f3. Two ascoids from f1 to f13. The internal ascoid is implanted approximatively at the same level as the external one. One terminal papilla on f1 and f2. No papilla from f3 to f9. One papilla on f10 and f11. Three papillae on f12 and four on f13 and f14. No simple setae observed from f1 to f12; three on f13 and eight on f14; Palpal formula: 1, (2, 3), 4, 5. About 15 basal Newstead’s sensilla on p3. No Newstead’s on p2. One distal spiniform seta on p3 and seven on p4, regularly implanted.

Mouth parts difficult to observe due to a lateral orientation of the holotype. Labrum 195 μm long. Maxillary lacinia with about 25 internal and 25 external teeth. Labial furca not observed; Cibarium with a horizontal row of 52 teeth arranged in palisade. Lateral ones are shorter than the others. We observe just below 21 small punctate vertical teeth linked to them. In the middle of the pigment patch, there is a transverse line of 19 vertical teeth of dot-like appearance. The pigment patch is decomposed into a rather pigmented, sausage-shaped posterior part, crossing the entire cibarium. In the anterior part, there is a little pigmented triangle. The pharynx is armed at its posterior third by many strong teeth oriented backwards.

##### Cervix

Cervical sensilla and ventro-cervical sensilla not observed.

##### Thorax

About 460 μm long, dark sclerites, absence of post-alar setae, paratergital setae on the pleural sclerites not observed, absence of proepimeral setae, absence of upper anepisternal setae, absence of lower anepisternal setae, absence of anepimeral setae, absence of metepisternal setae, absence of metepimeral setae, presence of setae on the anterior region of the katepisternum. Suture between metepisternum and katepimeron not observed. Metafurca mounted laterally; it is not possible to see clearly whether the vertical arms are joined by a membrane or not.

##### Wing

Length: 1888 μm; width: 482 μm. R5 = 1298 μm; alpha (=R2) = 268 μm; beta (=R2 + 3) = 375 μm; gamma (=R2 + 3 + 4) = 327 μm; delta = 90 μm; pi = 165 μm; epsilon (=R3) = 445 μm; theta (=R4) = 921 μm.

##### Legs

Anterior leg: coxa = 329 μm; femur = 664 μm; tibia = 728 μm; tarsomere i = 357 μm; sum of tii, tiii, tiv, tv = 603 μm. Median leg: coxa = 297 μm; femur = 648 μm; tibia = 888 μm; tarsomere i = 437 μm; sum of tii, tiii, tiv, tv = 650 μm. Posterior leg: coxa = 307 μm; femur = 728 μm; tibia = 1044 μm; tarsomere i = 532 μm; sum of tii, tiii, tiv, tv = 724 μm. Absence of spines on the metafemur. One verticil of spines in the middle of the metatarsomere iii.

##### Abdomen

Setae on tergites ii–v not observed. Tergite VIII, IX and sternite IX not observed.

##### Genitalia

Body of the spermathecae finely wrinkled with an enlargement at the base. One spur at the top, close to the knob. The terminal knob is embedded in the body and carries about 15 canaliculi. Spermathecal ducts thin and wide with a basal common duct as long as the individual ones. After mounting, the spermathecal ducts were not observable and the aspect of the spermathecal body changed. Cerci not observed.

### *Riouxomyia* Depaquit, Blavier & Randrianambinintsoa subg. nov.

urn:lsid:zoobank.org:act:60D5FC67-4656-4EBF-866E-1EB677E97BE8

Based on female: presence of two ascoids on flagellomeres f1–f13 (=AIII–AXV); absence of setae on the following sclerites of the thorax: proepimeron, anepisternum, anepimeron, katepimeron, metepisternum and metepimeron; spermathecae finely wrinkled with an enlargement at the base and one spur at the top, close to the knob.

Type-species: *Sergentomyia (Riouxomyia) kaltenbachi* Depaquit, Blavier & Randrianambinintsoa n. sp.

Etymology: the subgenus is dedicated to the memory of our colleague Jean-Antoine Rioux.

## Discussion

The research carried out in Ankarana involved the collection of 723 phlebotomine sandflies. The material studied included nine species, this richness being the highest documented at any single location in Madagascar. This inventory confirms that *tsingy* areas are favorable environments for phlebotomine sandflies. Caves associated with constant temperatures and humidity are presumed to be excellent resting places as compared to other areas [35].

*Phlebotomus fertei* is the only species of the genus *Phlebotomus* recorded at Ankarana. It is relatively abundant (120 specimens representing about 17% of those captured), and remains the most widespread and studied *Phlebotomus* in Madagascar. The specimens from Ankarana are morphologically similar to the type series from Bemaraha, also a *tsingy* habitat.

In the material we collected in Ankarana, genus *Grassomyia* was presented by four specimens, three males and one female. Considering that the systematics of this group are probably among the most complicated regarding Phlebotomine sandflies, we did not go into considerable detail. Males are much more difficult to identify than females and out of four specimens of genus *Grassomyia* captured, only one was a female. Although the only female specimen might belong to *Gr. madagascariensis*, due to the presence of 40 cibarial teeth, the species identification of the males remains doubtful. This genus is poorly represented at Ankarana and is much more frequent in open landscapes elsewhere in the country. The record of *Gr. squamipleuris* in Madagascar [34] calls for confirmation, as the type species is from continental Africa (Khartoum, Sudan) and all other phlebotomine sandflies on Madagascar are endemic.

**Figure 16:**
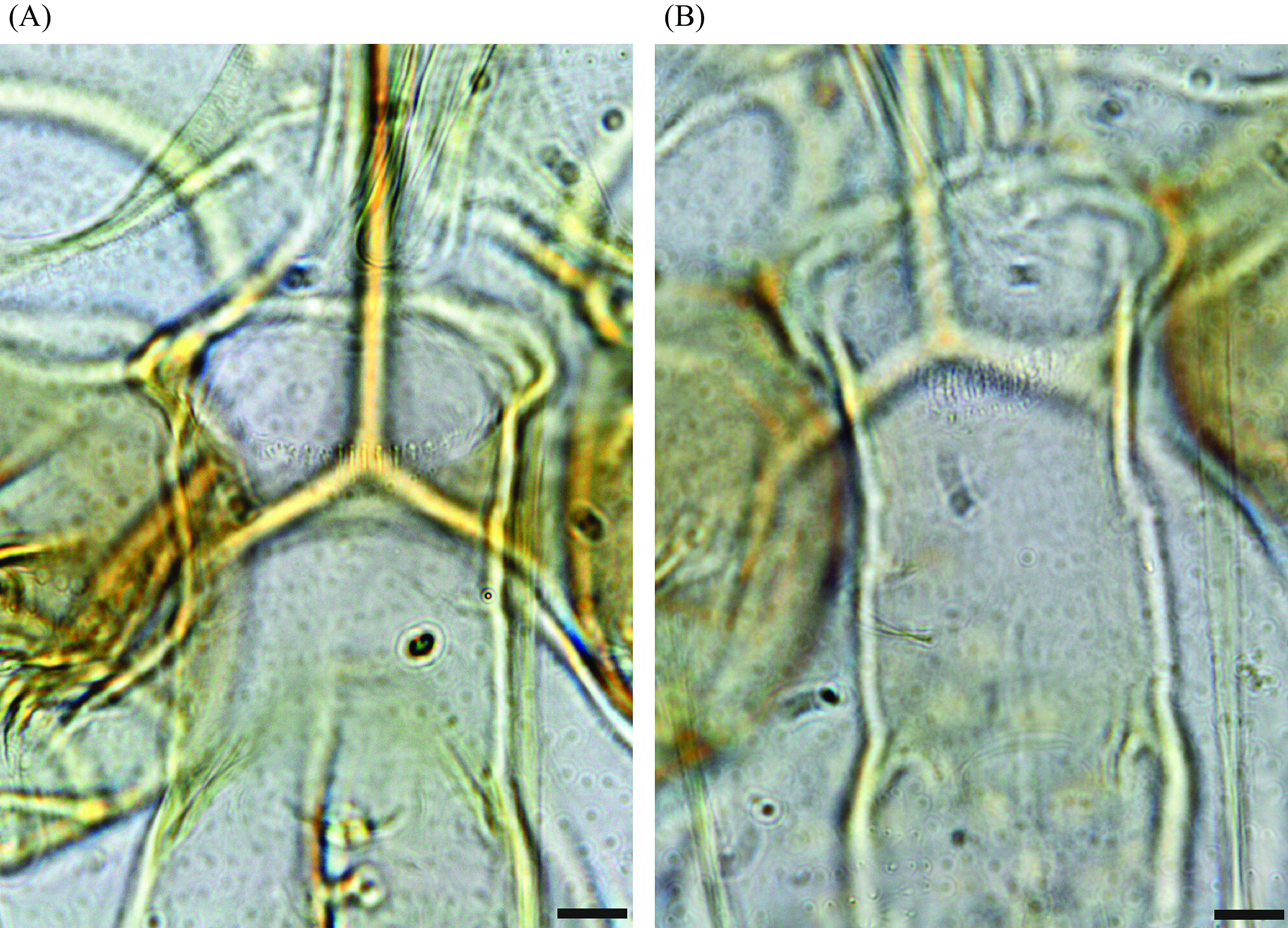
Cibarial armature of males of *Sergentomyia sclerosiphon* (A) and *Se. anka* (B). Bars = 10 μm.

In this study, we focused mainly on the genus *Sergentomyia*, which was the most abundant in our study representing over 82% of the captures, despite a controversial role in the transmission of *Leishmania* spp. [28]. The species most frequently caught belonged to the subgenus *Vattieromyia* which accounted for more than 75% of the individuals identified. This subgenus consists of four previously described species: *Se. sclerosiphon* and *Se. anka* described from Ankarana [12], *Se. namo* described from Namoroka [12], and *Se. pessoni* described from the Comoros Archipelago [31]. Males and females have already been described for the first two species. For two other species, only females are known. As has frequently been observed in the genus *Sergentomyia*, the species diagnosis is simpler for females than for males. The latter are sometimes morphologically very similar, which has complicated their identification, especially for *Vattieromyia*. Indeed, the position of the accessory spine on the gonostyle is difficult to evaluate and there is some overlap in the length measurements of the flagellomeres. Moreover, the cibarial armature of males is discrete and depends on the condition of the specimen, including how it is positioned on the slide, and the use of phase contrast microscopy (Fig. 16).

We identified the Ankarana material based on comparison with type specimens. In many cases, the spermathecae are the primary character for subgeneric classification of sandflies, but their use at the species level is limited. It is often easier to identify species based on the teeth of the cibarium. For *Se. anka*, the original description is based on the presence of a posterior row of 35–43 teeth (average: 38). The central ones are much longer than the lateral ones and are curved. Based on a larger sample than in the original description, we found different values: from 30 to 45 with an average of 37.7. Regarding the number of vertical teeth in the first row, we counted from 5 to 11 teeth (against 7–10 in the original description). Finally, we found in all of our samples, excepting one (MADA817), the second row of vertical teeth, which as in the original description, were within the range from 3 to 7. In our material, the number of anterior vertical teeth varied from 1 to 7.

The identification of *Se. sclerosiphon* females is mainly based on more widely spaced cibarial teeth with straight central ones. We counted 21–34 teeth versus 20–29 in the original description, and 7–22 vertical teeth in the first row versus 8–15 in the original description. A second row of vertical teeth was not mentioned in the original description, but we found this additional row in 66 specimens out of the 218 examined. The number of vertical teeth constituting this most anterior row varies from 1 to 15, but ranged between 8 and 15 in most of the specimens. In males, the nearly distal position of the accessory spine of the gonostyle does not seem to be very useful for their identification. Similarly, the length of the first flagellomere is of no use for species identification, as there is an overlap between two species: in *Se. anka*, the length varies from 158 to 248 μm and in *Se. sclerosiphon*, from 179 to 287 μm. Consequently, if the length of the first flagellomere is less than 179 μm or longer than 248 μm, identification is possible. Finally, our morphological identifications were based exclusively on the cibarial armature.

**Figure 17:**
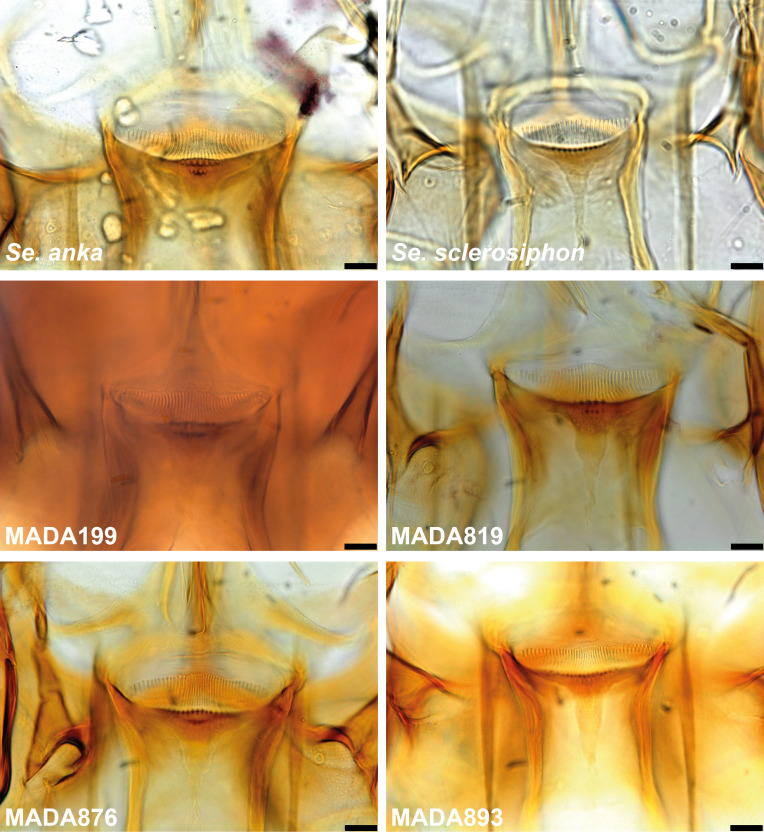
Comparison of the cibarial armatures of *Sergentomyia anka* and *Se. sclerosiphon* females with those of specimens identified as *Se.* cf. *anka* and isolated by molecular analyses. Bars = 10 μm.

The molecular approach highlights the difficulties involved with *Vattieromyia* taxonomy. The morphological approach is generally supported by the sequences of cytochrome b that classified both sexes into two branches for the majority of *Se. anka* and *Se. sclerosiphon* specimens. On the other hand, the D1 and D2 domains of the rDNA were insufficient to differentiate these two species. This observation highlights the conservation of this marker versus the great variability of cytochrome b. Some specimens are isolated: MADA 95 (♂), MADA96 (♂), MADA199 (♀), MADA819 (♀), MADA876 (♀), MADA893 (♀), MADA897 (♂), MADA898 (♂) and MADA 1345 (♂). They draw our attention during their morphological examination due to their less typical cibarial armatures (Fig. 17). We carefully identified them as *Se. cf anka*. It is very interesting to note that both Cytb and D1 and D2 sequences clearly individualize these specimens. The genetic distance between these atypical specimens is 2.5% for cyt b and 0.4% for D1 and D2. This is much higher than within other species. It is also much higher between these individuals than within true *Se. anka* or *Se. sclerosiphon*, for which the variability observed is only of 0.5% and 0.3%, respectively (Tables 3 and 4). This genetic distance is close to the mean genetic distance between true *Se. anka* and *Se. sclerosiphon*. Nevertheless, we were not able to provide more definitive identification of these atypical specimens and they should not be considered a subspecific form due to their sympatry with *Se. anka* and *Se. sclerosiphon*. It is possible that they represent some form of differentiation within Ankarana, but there is no strong morphological or molecular evidence to support their recognition as a new species and until further data are available, we consider these specimens atypical.

Morphological and molecular evidence supports the recognition of a new species: *Se. volfi* n. sp. The spermathecae have a slightly wrinkled structure, without sclerotization, characters unknown in any named subgenus. The third segment of the palp is particularly wide in the female and covered with a large number of Newstead’s spines. Moreover, the absence of the ascoid on flagellomeres 1 and 2 in both males and females is seemingly unique in phlebotomine sandflies. The latter character, associated with the particular structure of the spermatheca, justify a new subgenus: *Ranavalonomyia* subg. nov. We also observed a unique distal fringe on top of flagellomeres f1–f6 in both females and males.

**Figure 18:**
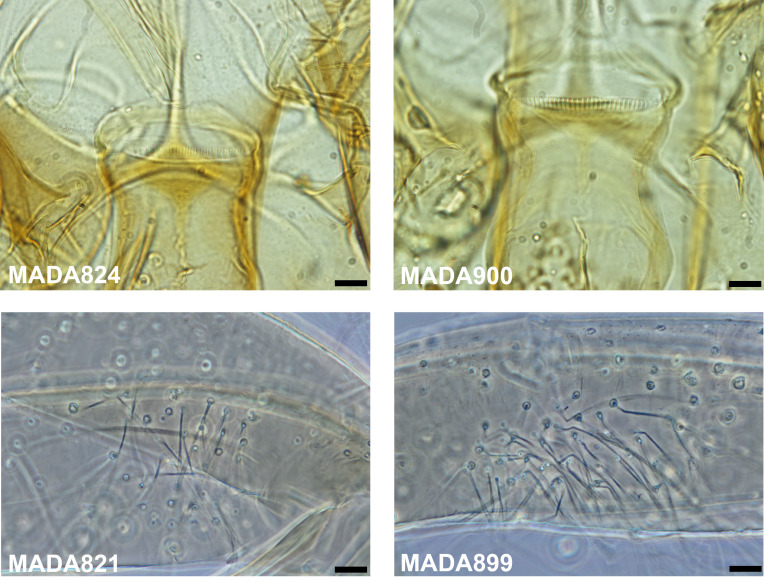
*Sergentomyia* (Ranavalonomyia) *volfi* n. sp. Cibarial armatures of two females exhibiting 20 (MADA824) or 30 (MADA900) teeth and coxites of two males exhibiting 9 (MADA821) and 23 setae (MADA899). Bars = 10 μm.

Molecular data show a high degree of intraspecific variability (1.6% with cyt b), which is related to morphological characters (Fig. 18), and seems to indicate two possible distinct populations. Regarding females, a wide range of cibarial teeth (20–31) was noted in our limited sampling (*p* = 0.51, not statistically significant, passing the normality test of D’Agostino & Pearson). For males, the number of gonocoxal setae varied from 9 to 24 (*p* = 0.73, not statistically significant, passing the normality test of D’Agostino & Pearson). To our knowledge, this is a greater degree of intraspecific variability than has previously been observed in other species of phlebotomine sandflies. The morphological variables passed the D’Agostino & Pearson normality test. Without any greater evidence, we consider that all these specimens belong to the same species.

The association of males and females is strongly supported by their sequence homology.

The classification of *Se*. *ozbeli* n. sp. in the subgenus *Rondanomyia* is based on its spermatheca, which is distinctive in this group. *Sergentomyia goodmani* from Madagascar and its subspecies *Se. goodmani comorensis* from the Comoros do not have any cibarial vertical teeth. The identification of *Se*. *ozbeli* n. sp. is based on the existence of two rows of cibarial vertical teeth, the most anterior ones forming a continuous line completely crossing the sclerotized area.

*Sergentomyia kaltenbachi* n. sp. is very different from the other Malagasy species. It has a cibarium with a very large number of teeth in the form of a palisade and a morphologically distinct spermatheca. The spermathecal body wall is finely wrinkled and exhibits a lateral spur, with a head embedded in the body. Considering the uniqueness of its spermatheca, we propose a new subgenus for this species: *Riouxomyia* subg. nov.
